# Harnessing Guanidinium and Imidazole Functional Groups: A Dual-Charged Polymer Strategy for Enhanced Gene Delivery

**DOI:** 10.1021/acsmacrolett.4c00321

**Published:** 2024-07-25

**Authors:** Prosper P. Mapfumo, Liên S. Reichel, Katharina Leer, Jan Egger, Andreas Dzierza, Kalina Peneva, Dagmar Fischer, Anja Traeger

**Affiliations:** † Institute of Organic Chemistry and Macromolecular Chemistry (IOMC), Friedrich Schiller University Jena, Humboldtstrasse 10, 07743 Jena, Germany; § Jena Center for Soft Matter (JCSM), Friedrich Schiller University Jena, Philosophenweg 7, 07743 Jena, Germany; ∥ Center for Energy and Environmental Chemistry Jena (CEEC), Friedrich Schiller University Jena, Philosophenweg 7, 07743 Jena, Germany; ⊥ Division of Pharmaceutical Technology and Biopharmacy, Friedrich-Alexander-Universität Erlangen-Nürnberg, Cauerstr. 4, 91058 Erlangen, Germany; # FAU NeW - Research Center New Bioactive Compounds, Friedrich-Alexander-Universität Erlangen-Nürnberg, Nikolaus-Fiebiger-Str. 10, 91058 Erlangen, Germany

## Abstract

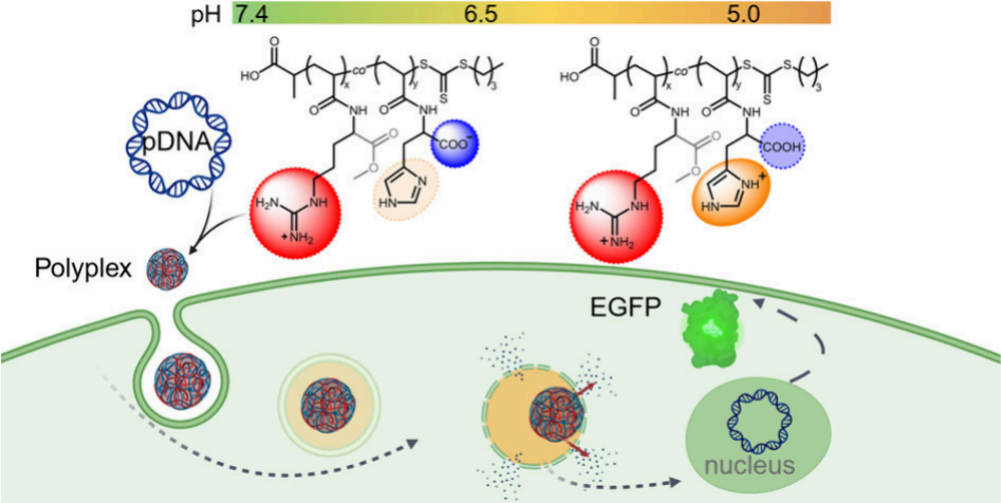

Histidine and arginine are two amino acids that exhibit beneficial properties for gene delivery. In particular, the imidazole group of histidine facilitates endosomal release, while the guanidinium group of arginine promotes cellular entry. Consequently, a dual-charged copolymer library based on these amino acids was synthesized via reversible addition–fragmentation chain transfer (RAFT) polymerization. The content of the *N*-acryloyl-l-histidine (His) monomer was systematically increased, while maintaining consistent levels of methyl *N*-acryloyl-l-argininate hydrochloride (ArgOMe) or *N*-(4-guanidinobutyl)acrylamide hydrochloride (GBAm). The resulting polymers formed stable, nanosized polyplexes when complexed with nucleic acids. Remarkably, candidates with increased His content exhibited reduced cytotoxicity profiles and enhanced transfection efficiency, particularly retaining this performance level at lower pDNA concentrations. Furthermore, endosomal release studies revealed that increased His content improved endosomal release, while ArgOMe improved cellular entry. These findings underscore the potential of customized dual-charged copolymers and the synergistic effects of His and ArgOMe/GBAm in enhancing gene delivery.

Gene delivery plays a pivotal role in gene therapy, enabling therapeutic approaches in target cells.^1^ The potential benefits encompass genetic defect correction, gene expression modulation, and enhanced cellular functions.^1−3^ Despite its significance, gene delivery encounters challenges, necessitating safe and efficient carriers to protect genetic material, prevent degradation, and ensure successful uptake, endosomal release, and gene modulation.^4−6^ Synthetic polymers are promising gene therapy carriers due to their versatile structural design and ease of large-scale production, among other factors.^7,8^

Arginine, an amino acid, shows immense potential in gene delivery due to its guanidinium functional group.^9−13^ This functional group induces temporary pore formation in cell membranes, facilitating efficient genetic cargo transport.^14,15^ Our previous research on homopolymers with various amino functional groups as gene carriers demonstrated that an increased number of guanidinium groups leads to more efficient cellular uptake and endosomal release.^16^ However, although the positively charged guanidinium functional group improves gene delivery, it also aids increased cytotoxicity.^16,17^ To address charge-related toxicity, integration of anionic polymers has shown potential.^18−20^ Therefore, dual-charged and zwitterionic polymers are of particular interest and are increasingly recognized as potential carriers for drug and gene delivery.^20−22^ By incorporating both positive and negative charges within a polymer, the overall positive charge is reduced, leading to a decrease in cytotoxic effects.^19^ For example, Kim et al. demonstrated a superior reduction in the cytotoxicity of micelles, incorporating zwitterionic arginine compared to their cationic methylated arginine counterparts.^23^ In addition to the cytotoxicity benefits, these dual-charged polymers possess low fouling characteristics, reduced protein adsorption, and prolonged blood circulation.^24−27^ Furthermore, their dynamic pH-responsiveness enhances interactions with cell membranes, aiding cellular uptake and cytoplasmic genetic material release.^28−30^

To this end, histidine, a pH-responsive amino acid, exhibits considerable potential in delivery carriers.^31^ Its imidazole group buffers the endosomal acidic environment, destabilizing membranes and facilitating the rapid release of nanoparticles or complexes into the cytosol.^32−34^ As highlighted by Hooshmand et al., modified polymers and peptides incorporating histidine display enhanced endosomal release and gene transfection efficacy, thus presenting a promising avenue for enhancing gene therapy outcomes.^31^ While researchers have focused on the imidazole group, there have been few investigations into the zwitterionic form, albeit its benefits. For example, Bertrand et al. explored the incorporation of histidine involving both free imidazole and carboxylic groups. Their findings demonstrated a significant increase in transfection efficiency along with reduced cytotoxicity.^35^

This study aims to harness the cell-penetrating and endosomal release capabilities of guanidinium and imidazole groups. A copolymer library containing monomers derived from histidine (*N*-acryloyl-l-histidine or His) and two based on arginine, methyl *N*-acryloyl-l-argininate hydrochloride (ArgOMe) and *N*-(4-guanidinobutyl)acrylamide hydrochloride (GBAm), was synthesized via RAFT polymerization. In this newly designed dual-charged copolymer library, His comprises a carboxylic acid moiety and a pH-dependent protonatable imidazole group, while ArgOMe and GBAm consist of the cationic guanidinium moiety. After comprehensive chemical characterization, the copolymer library’s potential as a gene carrier was evaluated, focusing on parameters such as transfection efficiency, cytotoxicity, and endosomal release.

*Synthesis and characterization*: First, ArgOMe, His, and GBAm, consisting of a butyl spacer analogous to ArgOMe, were synthesized via nucleophilic addition–elimination type reaction (Figure S1).^35−37^ The monomers were subsequently polymerized using RAFT polymerization, as depicted in Figure 1A. This approach is chosen for its versatility and compatibility with monomers possessing unprotected functionalities and a broad range of reaction conditions.^38,39^ The polymers were designed to ensure that ArgOMe/GBAm is responsible for binding to genetic material. Meanwhile, His predominantly assumes an anionic charge at physiological pH and becomes cationic at endosomal pH (5.5),^40^ thus, resulting in pH-responsive dual-charged polymers with reduced net positive charges. While zwitterionic and histidine-derived polymers have been studied,^21,31^ the specific combination of the designed copolymers has not been explored before.

**Figure 1:**
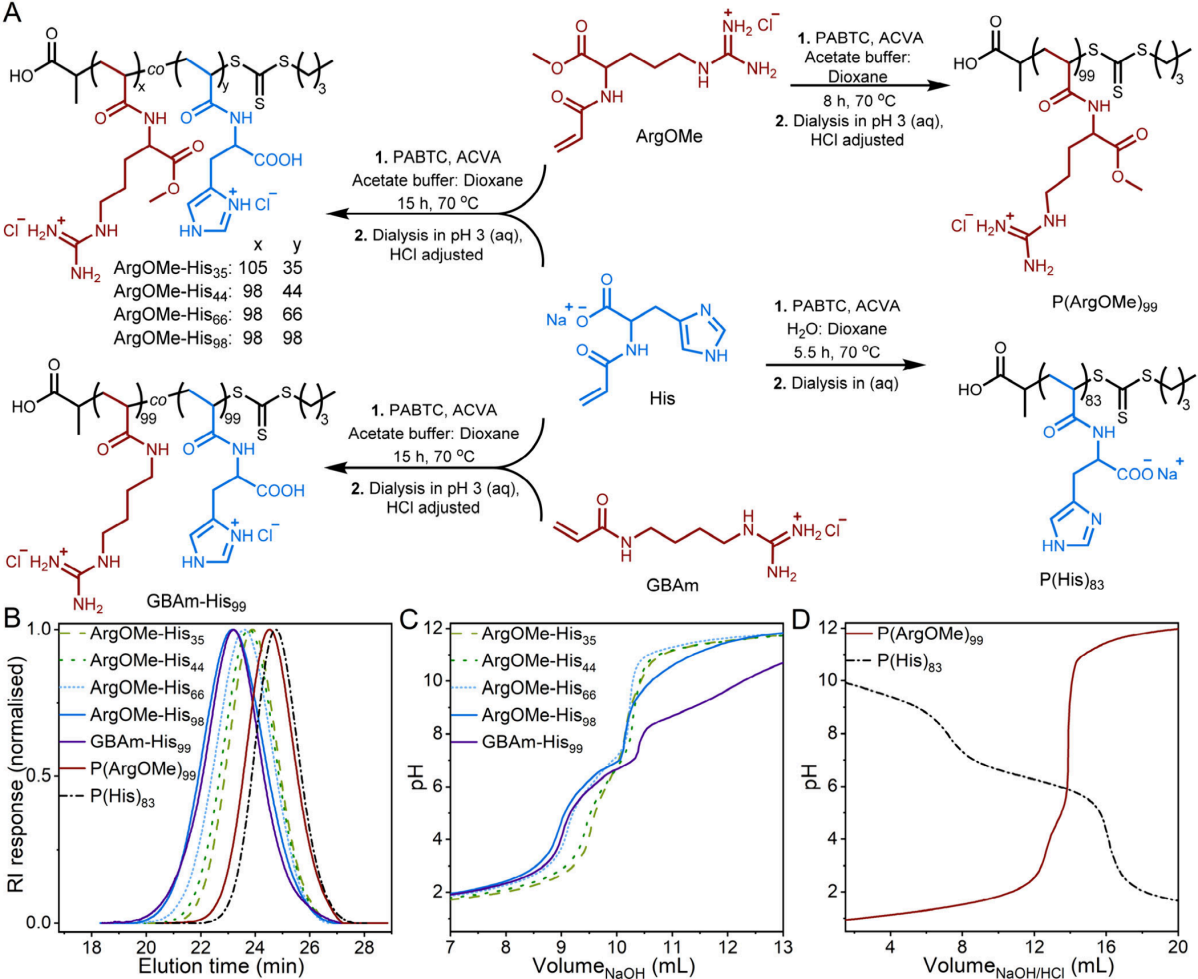
(A) Reaction scheme illustrates the structures of the monomers and polymer library as well as their polymerization conditions. (B) SEC traces of the polymers determined using water (+0.1% TFA and 0.1 M NaCl) as the eluent. (C, D) pH titration curves were determined using 0.15 M NaOH for all polymers except P(His)_83_, which was determined using 0.2 M HCl.

Initially, a kinetic reaction was performed to establish the reaction conditions and monitor polymerization control (Figure S2). The monomers showed similar reactivity, but a nonlinear semilogarithmic plot suggested a decrease in active propagating species over time, likely due to termination reactions.^41^ Afterward, polymers P[(ArgOMe)_105/98_-*co*-(His)_35/44/66/98_] (denoted as ArgOMe-His_35/44/66/98_) were synthesized with varying compositions, i.e., systematically increasing the amount of His while maintaining a relatively constant amount of ArgOMe. This aimed to evaluate the impact of increasing His content on polymer properties and biological applications. To assess the influence of the methyl ester functional group, ArgOMe was replaced with GBAm to synthesize P[(GBAm)_99_-*co*-(His)_99_] (denoted as GBAm-His_99_), an analogue to ArgOMe-His_98_. To study individual components in isolation, P(ArgOMe)_99_ and P(His)_83_ were synthesized. Finally, two additional controls were synthesized: P(GBAm)_88_ and P[(GBAm)_102_-*co*-(His)_51_] (denoted as GBAm-His_51_). The latter was selected as an intermediate of ArgOMe-His_35/44/66_ to assess the biological effects of combining GBAm with a low molar content of His. As shown in Figure 1B, SEC traces displayed monomodal molar mass distributions for all polymers (further details are provided in the SI, Table S1 and Figures S3–S6). Moreover, Table 1 reveals that the synthesis exhibited good polymerization control, as indicated by relatively low dispersities (*Đ*) below 1.5 for all polymers.

**Table 1:** Summary of Molar Masses, Apparent p*K*_a_ Values and IC_50_ of the Polymer Library

	ArgOMe-His_35_	ArgOMe-His_44_	ArgOMe-His_66_	ArgOMe-His_98_	GBAm-His_99_	P(ArgOMe)_99_	P(His)_83_
*M*_n,th_^a^ (kg mol^–1^)	38.0	38.3	43.8	51.4	46.4	27.9	19.5
*M*_n,SEC_^b^ (kg mol^–1^)	13.2	14.1	15.7	18.2	18.3	8.6	5.7
*Đ*^b^	1.3	1.3	1.4	1.4	1.4	1.4	1.5
p*K*_a_^c^ (His)	6.1	6.1	6.2	6.4	6.4		6.3
IC_50_^d^ (μg mL^–1^)	79.8	88.1	102.3	105.2	99.1	46.0	

a Calculated via conversion using ^1^H NMR (eq S1).

b Determined by SEC using water (+0.1% TFA and 0.1 M NaCl) as eluent and P2VP standards for calibration.

c Calculated by eq S2 and determined by Figure S7.

d IC_50_ calculation was done with DoseRespond fit function using OriginePro Software (Version 2022b).

Afterward, the pH responsiveness of the polymers was assessed by titrations. As expected, an increase in the buffering region between pH 5 and 7 due to the increase in the imidazole group was observed (Figure 1C). This was notable when comparing ArgOMe-His_35_ and ArgOMe-His_98_. However, the apparent p*K*_a_ values of His for ArgOMe-His_35/44/66/98_ and GBAm-His_98_ were comparable, ranging between 6.1 and 6.4, as shown in Table 1. These values were similar to that of P(His)_83_, which was 6.3, all of which were closely related to the p*K*_a_ of histidine, approximately 6.0.^31,35^ In contrast, the guanidinium group was assumed to have a p*K*_a_ above pH 11 since it has as a p*K*_a_ of 13.8 in arginine and no distinct plateau was observed for P(ArgOMe)_99_ (Figure 1D).^42^ It is worth noting that all polymers except P(His)_83_ precipitated during the titrations. Specifically, ArgOMe-His_35_, ArgOMe-His_44_, and P(ArgOMe)_99_ precipitated at pH values above 10, while ArgOMe-His_66_, ArgOMe-His_98_, and GBAm-His_99_ precipitated between pH 6.8 and 7.5. This behavior was attributed to the polymers becoming more neutrally charged during titration. Similarly, Leiske and co-workers observed aggregation in dual-charged polymers at a neutral charge.^22^

*Polyplex characterization*: As shown in Figure 2, the ability of the polymers to bind pDNA was assessed qualitatively and quantitatively using horizontal agarose gel electrophoresis and a fluorophore dye exclusion assay, respectively.

**Figure 2:**
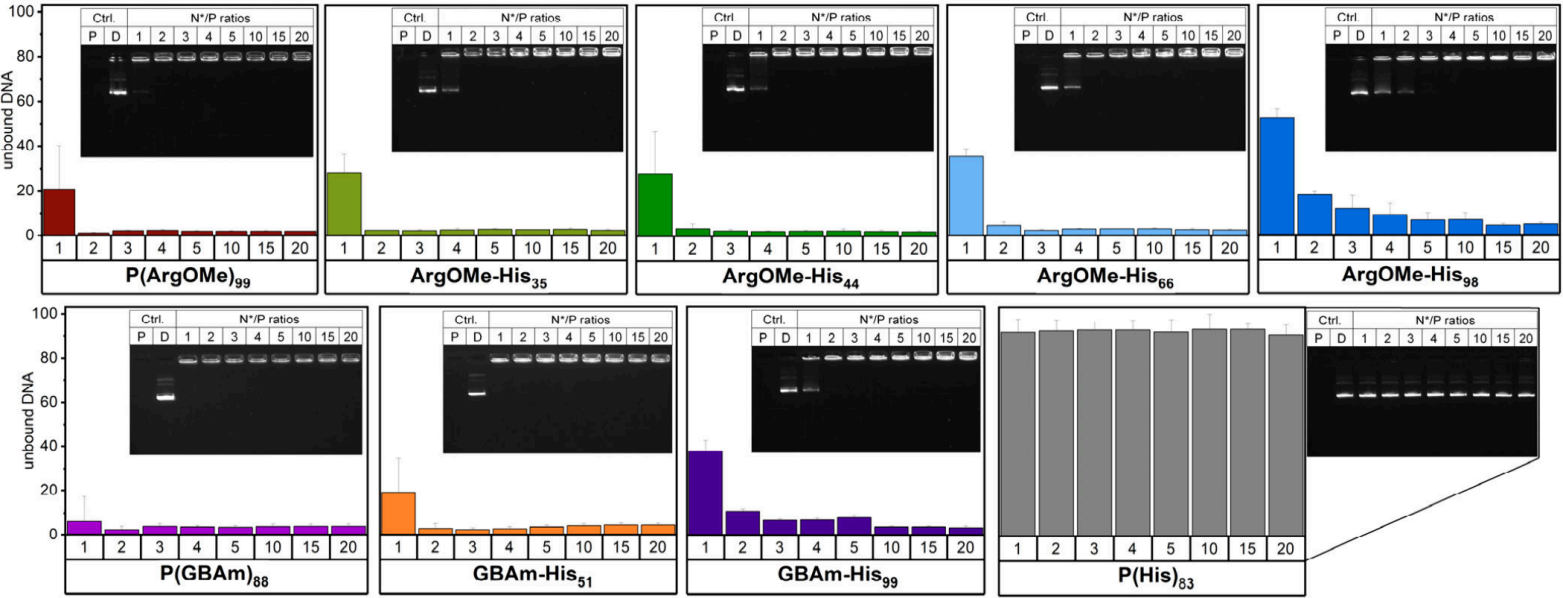
pDNA binding efficiency of the polymers was determined with the AccuBlue High Sensitivity dsDNA Quantitation Kit (bottom), and horizontal agarose gel electrophoresis (top), with free polymer (P) and free DNA (D) as controls.

The polymers ArgOMe-His_35_, ArgOMe-His_44_, ArgOMe-His_66_, and P(ArgOMe)_99_ showed an almost complete binding of pDNA starting at a ratio of N*/P 2 (where N*/P is protonatable nitrogens in the polymer to phosphates in the pDNA). In contrast, ArgOMe-His_98_ and GBAm-His_99_ exhibited a more pronounced N*/P ratio-dependent binding behavior with increased binding affinities for rising N*/P ratios. P(His)_83_ did not result in complexation of pDNA, regardless of the N*/P ratio, due to the absence of the guanidinium group, indicating that His alone is not sufficient for the interaction with pDNA. This characteristic is due to the low p*K*_a_ of the imidazole group in P(His)_83_, rendering the polymer predominantly anionic at physiological pH levels. To this end, higher molar ratios of His compared to ArgOMe/GBAm result in an overall negative charge of the polymer, potentially resulting in poor binding.

*Cytotoxicity and polymer–membrane interaction*: Cytotoxicity profiles of the polymers were assessed using PrestoBlue assay in the mouse fibroblast cell line L929, following ISO10993-5 guideline.^43^ The PrestoBlue assay is a sensitive resazurin-based method that measures the relative metabolic activity of viable cells. Results are shown in Figure 3 and the IC_50_ values are displayed in Table 1. Notably, P(His)_83_ demonstrated nontoxicity within the tested range. Conversely, P(ArgOMe)_99_, P(GBAm)_88_, and GBAm-His_51_ displayed higher toxicity, as indicated by low IC_50_ values of 46.0, 39.7, and 49.0 μg mL^–1^, respectively. However, the trends observed for the His-containing copolymers showed an intriguing pattern: an increase in His content and consequently a dual-charged character led to improved cell viability. These findings underscore how incorporating His into P(ArgOMe) or P(GBAm) results in a dual-charged copolymer with reduced net positive charges, thereby mitigating cytotoxicity. This is aligned with the findings of Kim et al., which showed that zwitterionic arginine micelles were nontoxic compared to their cationic counterparts.^23^

**Figure 3:**
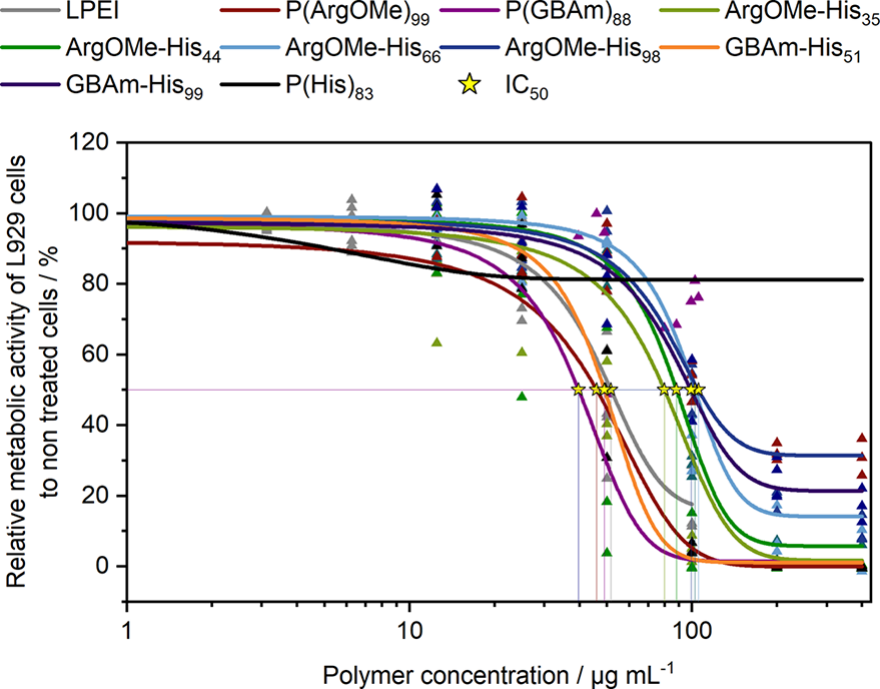
PrestoBlue assay was performed for 24 h in L929 cells. Dots represent values of single repetitions (*n* ≥ 3), and lines represent DoseRespond fit function. Stars indicate 50% lethal polymer concentration (IC_50_).

*Transfection efficiency*: Subsequently, a transfection efficiency assay was conducted for all polymers using the human embryonic kidney cell line HEK293T over 24 h. The cells were cultured in Dulbecco’s modified Eagle’s medium supplemented with 10% serum and 1% 4-(2-hydroxyethyl)-1-piperazineethanesulfonic acid (D10H). Linear polyethylenimine (LPEI) was included as an experimental positive control. The polyplexes were evaluated at N*/P 15, except LPEI at N*/P 20. Dynamic light scattering (DLS) results revealed that the sizes of the polyplexes were below 100 nm for all guanidinium functionalized polymers, indicating binding and complexation of all polymers to sizes suitable for endocytotic uptake (Tables S2 and S3 and Figure S8).^44^ As expected, P(His)_83_ displayed a broad polydispersity index (PDI) of 0.9 due to its inability to bind pDNA, as showcased in Figure 2.

Conversely, guanidinium-functionalized polymers exhibited monomodal intensity-weighted size distributions with PDIs below 0.2 and demonstrated high stability when assessed after 40 days of storage at 4 °C (Table S2). Moreover, increased His content improved polyplex stability under acidic conditions (Table S3). The transfection performances of the polyplexes were tested across four distinct pDNA concentrations. At the highest concentration of pDNA (3 μg mL^–1^), supernatant samples were taken to perform the CytoTox-ONE assay. This assay relies on a fluorometric method akin to PrestoBlue, and it is utilized to assess cell viability by analyzing membrane integrity. Figure 4 illustrates the outcomes, revealing that the incubation of the polyplexes resulted in minimal cytotoxicity, with over 90% viability, except for P(ArgOMe)_99_ and P(GBAm)_88_. Their toxic nature was notable, with viability reducing to approximately 80%. In terms of transfection efficiencies, assessed by quantifying the proportion of viable single cells expressing enhanced green fluorescent protein (EGFP), intriguing trends were observed (Figure 4). At the highest pDNA concentration of 3 μg mL^–1^, LPEI, ArgOMe-His_35/44/66_, and GBAm-His_51_ showed comparable transfection performance, with the latter displaying slightly improved performance.

**Figure 4:**
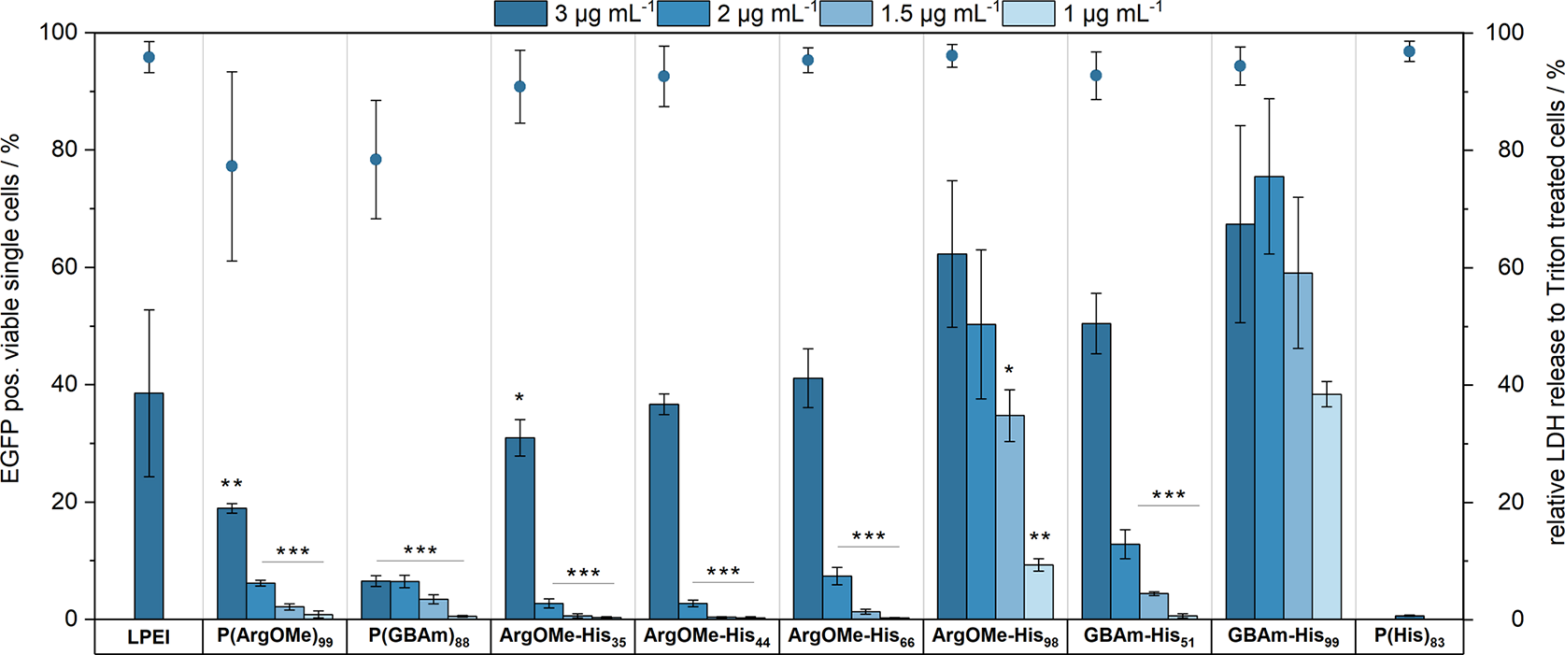
Dots represent viability conducted via CytoTox-ONE assay (*n* = 3) at 3 μg mL^–1^ of mEGFP-N1 pDNA on cells. Bars represent transfection efficiency assay conducted in full growth medium at N*/P 15 with different pDNA concentrations in HEK293T cells over 24 h (*n* ≥ 3). LPEI at N*/P 20 and 3 μg mL^–1^ of mEGFP-N1 pDNA on cells was used as control. Significancy of ArgOMe-His_35/44/66/98_ and P(ArgOMe)_99_ to GBAm-His_99_ at the respective pDNA concentrations are illustrated as p* > 0.05, p** > 0.01, and p*** > 0.001. Further results can be found in the SI (Figures S9–S11).

Conversely, P(His)_83_ displayed negligible transfection, which was to be expected due to its poor binding ability (Figure 2). Remarkably, ArgOMe-His_98_ and GBAm-His_99_ exhibited greater efficacy, surpassing the performance of the control, LPEI. Additionally, GBAm-His_99_ displayed significantly improved transfection efficiency compared to ArgOMe-His_35/44/66_, GBAm-His_51_ and the control polymers, P(ArgOMe)_99_ and P(GBAm)_88_. These findings underscore the crucial synergy between ArgOMe or GBAm and His in enhancing transfection efficiency, particularly through increasing the molar ratio of the latter. Moreover, this highlights the pivotal role of His in augmenting transfection performance and cytotoxicity profiles.

Discernible differences in performance were observed at lower pDNA concentrations. All polymers, except ArgOMe-His_98_ and GBAm-His_99,_ showed efficiencies below 20% at pDNA concentrations of 2, 1.5, and 1 μg mL^–1^. In contrast, ArgOMe-His_98_ and GBAm-His_99_ maintained relatively high efficiencies across the same pDNA concentration range, with a notable decline in performance observed only for ArgOMe-His_98_ at the lowest pDNA concentration. Intriguingly, at 1.5 and 1 μg mL^–1^ pDNA, GBAm-His_99_ significantly outperformed ArgOMe-His_98_ (p* > 0.05 and p*** > 0.01). Furthermore, GBAm-His_99_ showed similar efficacy at the lowest pDNA concentration (1 μg mL^–1^) compared to all other polymers, except ArgOMe-His_98_, at their highest pDNA concentration (3 μg mL^–1^). This accentuated the superior performance of GBAm-His_99_, which demonstrated improved transfection efficiency compared to ArgOMe-His_98_ across all tested pDNA concentrations, despite both possessing similar His content. In terms of mean fluorescence intensity (MFI; Figure S10), a comparable trend was observed for ArgOMe-His_98_, GBAm-His_99_, and the control polymers P(ArgOMe)_99_, P(GBAm)_88_ as for EGFP-positive, viable single cells. However, at 3 μg mL^–1^ pDNA, the MFIs of ArgOMe-His_35/44/66_ and GBAm-His_51_ were comparable to slightly higher than GBAm-His_99_, but they drastically declined at lower pDNA concentrations. This outcome underlines the superiority of ArgOMe-His_98_ and GBAm-His_99_ at low pDNA concentrations on the protein expression level.

Considering the previous results, GBAm-His_99_ is the most effective polymer within the library and thus as the lead component. From a chemical point of view, the main difference between GBAm-His_99_ and ArgOMe-His_98_ is the absence of the methyl ester functional group in GBAm-His_99_. The difference in performance was presumed to be due to the poor colloidal stability of ArgOMe-His_98_ resulting from its tendency to aggregate, potentially leading to the loss of genetic material in the presence of serum. Since serum typically reduces performance,^16^ the positive results obtained with GBAm-His_99_ and ArgOMe-His_98_ at low pDNA concentrations in the presence of serum demonstrate the potential of the materials for potential *in vivo* applications. Additionally, the capacity of GBAm-His_99_ and ArgOMe-His_98_ to achieve high transfection efficiencies at lower polymer concentrations (N*/P 10, Figure S11) in comparison to P(ArgOMe)_99_ highlights the effectiveness of these polymers. This aspect is particularly crucial in maintaining improved biocompatibility.

*Endosomal release*: To study the impact of dual-charged polymers on membrane interaction, HEK293T cells were simultaneously incubated with a membrane nonpermeable dye (calcein) and polyplexes at N*/P 15 and 3 μg mL^–1^. As shown in Figure 5A, distinct green fluorescence dots indicate endocytotic uptake of calcein within cellular compartments, and diffuse green fluorescence pattern indicates endosomal calcein release, both of which were triggered by corresponding polyplexes. P(ArgOMe)_99_ revealed the highest fluorescence intensity, while an increase in His content between ArgOMe-His_35/44/66/98_ and GBAm-His_99_ resulted in a decrease in the fluorescence. It is known that positively charged molecules influenced the fluorescence intensity of calcein.^45^

**Figure 5:**
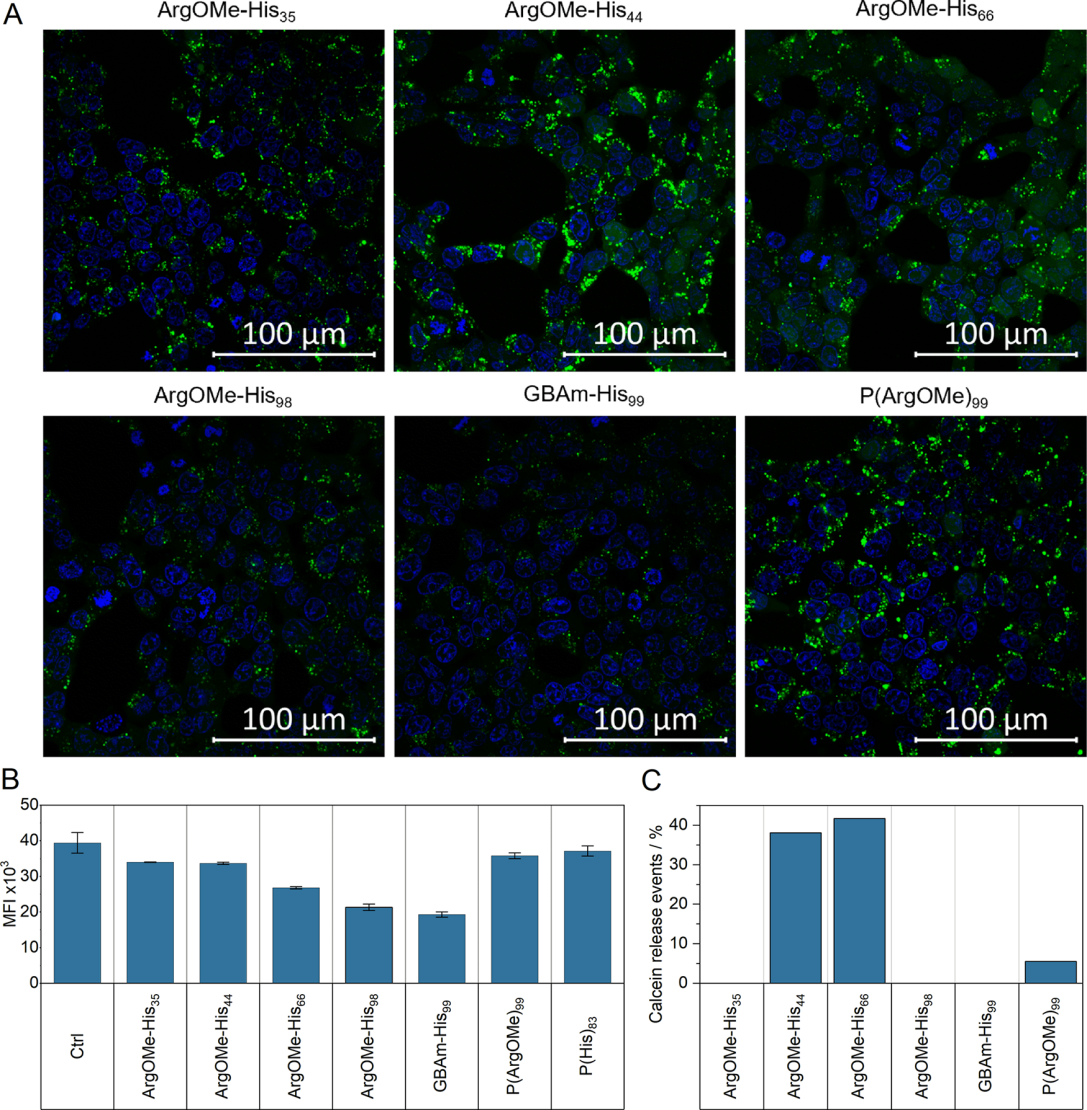
(A) Endosomal release was analyzed via confocal laser scanning microscopy (CLSM). HEK293T cells were simultaneously incubated with nonpermeable dye calcein (25 μg mL^–1^) and polyplexes (N*/P 15, 3 μg mL^–1^ pDNA) for 6 h. Green dots indicate endocytotic uptake of calcein within cellular compartments, and diffuse green fluorescence pattern indicates endosomal calcein release. The cell nuclei were stained with Hoechst 33342 (blue). More details can be found in the SI, Figures S12–14. (B) Fluorescence intensity of polymer and calcein after 6 h. 20 mM NaOAc buffer (pH 5.4) and HBG buffer (pH 7.4) with a mixing ratio of 1:1 (v/v) was used as control. The measurement was performed in triplicates (*n* = 3). (C) Calcein release event was analyzed using ImageJ version 1.54f after 6 h incubation.

To verify whether the decrease in intensity is correlated with lower endosomal uptake or due to the polymer–calcein interaction, additional investigation was conducted and revealed a decrease in fluorescence intensity by a higher His and lower guanidinium ratio (Figure 5B). However, all polyplexes induce a time-dependent endocytotic uptake and release (Figures S12–S14). Conversely, endosomal release shows an opposite trend, i.e., the increase in His content led to improved endosomal release events, thus P(ArgOMe)_99_ underperformed relative to His containing polymers. The high and detectable endosomal burst events of ArgOMe-His_44/66_ emphasize the critical role of histidine functionality (Figure 5C). Moreover, it can be observed that a suitable ratio of His and ArgOMe/GBAm is key in achieving optimal endosomal release at low endosomal pH value. Overall, the results are in good agreement with the significantly lower number of EGFP-positive cells of ArgOMe-His_35/44/66_ at 3 μg mL^–1^ pDNA in comparison to GBAm-His_99_.

In conclusion, preliminary findings identified P[(GBAm)_99_-*co*-(His)_99_] (GBAm-His_99_) and P[(ArgOMe)_98_-*co*-(His)_98_] (ArgOMe-His_98_) as the most promising candidates for safe and efficient pDNA delivery across various concentrations, addressing the challenge of balancing cytotoxicity with efficacy in gene therapy. The uptake attribute of guanidinium and the endosomal release of imidazole were successfully harnessed, albeit requiring the establishment of an optimal ratio for optimal performance. A high molar ratio of His resulted in improved endosomal release and, consequently, transfection performance. Cytotoxicity assessment revealed P(ArgOMe)_99_ and P(GBAm)_88_ as the most toxic among the polymers, while P(His)_83_ was nontoxic over the tested range. Consequently, P[(ArgOMe)-*co*-(His)] and P[(GBAm)-*co*-(His)] copolymers showed an improvement in cytotoxic profiles with increase in His content. Lastly, transfection efficiency investigations demonstrated the synergistic effect of ArgOMe/GBAm and His in improving performance, particularly at high molar ratios of the latter. Further investigations will focus on refining polymer properties such as solubility, a potential limiting factor of ArgOMe-His_98_, to fully unlock the potential of these polymers for *in vivo* applications.

## Supporting Information Available

Instruments; Materials; Synthesis and Characterization; Supporting Figures S1–S15 and Tables S1–S3; Supporting references (PDF)
