# Supplementary material for: Harnessing Guanidinium and Imidazole Functional Groups: A Dual-Charged Polymer Strategy for Enhanced Gene Delivery
Source: ACS Macro Lett. 2024 Jul 25;13(8):1000–7. doi: 10.1021/acsmacrolett.4c00321 (PMC11340021; doi:10.1021/acsmacrolett.4c00321)
Supplement: Supplementary file 1 — mz4c00321_si_001.pdf [file mz4c00321_si_001.pdf]

Supporting Information:

Harnessing Guanidinium and Imidazole Functional Groups: A Dual-Charged Polymer Strategy for Enhanced Gene Delivery

Prosper P. Mapfumo [a‡], *Liên S. Reichel* [a‡], *Katharina Leer* [a], *Jan Egger* [d], *Andreas Dzierza* [d], *Kalina Peneva* [a,b,c], *Dagmar Fischer* [b,d,e], *Anja Traeger* [a, b*]

[a] Institute of Organic Chemistry and Macromolecular Chemistry (IOMC), Friedrich Schiller University Jena, Humboldtstrasse 10, 07743 Jena, Germany

[b] Jena Center for Soft Matter (JCSM), Friedrich Schiller University Jena, Philosophenweg 7, 07743 Jena, Germany

[c] Center for Energy and Environmental Chemistry Jena (CEEC), Friedrich Schiller University Jena, Philosophenweg 7, 07743 Jena, Germany

[d] Division of Pharmaceutical Technology and Biopharmacy, Friedrich-Alexander-Universität Erlangen-Nürnberg, Cauerstr. 4, 91058 Erlangen, Germany

[e] FAU NeW - Research Center New Bioactive Compounds, Friedrich-Alexander-Universität Erlangen-Nürnberg, Nikolaus-Fiebiger-Str. 10, 91058 Germany

Instruments

Nuclear magnetic resonance (NMR) spectroscopy. ^{1}H NMR (300 MHz) and DEPT ^{13}C (75 MHz) spectra were recorded on a Bruker AC 300 MHz spectrometer at 300 K. The delay time (d1) was set at 1 s for ^{1}H NMR and 2 s for DEPT ^{13}C. Chemical shifts (δ) are reported in ppm.

Electrospray Ionization Quadrupole Time-of-Flight (ESI-Q-ToF). All samples were analyzed by using a microTOF Q-II (Bruker Daltonics) mass spectrometer equipped with an automatic syringe pump from KD Scientific for sample injection. The ESI-Q-ToF mass spectrometer was operating at 4.5 kV, at a desolvation temperature of 180 °C, in the positive (or negative) ion mode. Nitrogen was used as the nebulizer and drying gas. All fractions were injected using a constant flow rate (3 μL min^{-1}) of sample solution. The instrument was calibrated in the m/z range 50–3000 using a calibration standard (ESI-L Low Concentration Tuning Mix), which was supplied from Agilent Technologies. All data were processed via Bruker Data Analysis software version 4.2.

Size exclusion chromatography (SEC). Aqueous cationic-SEC was conducted using a Jasco instrument equipped with differential refractive index (DRI) and UV/vis (DAD) detector. The liquid chromatography system used PSS NOVEMA-MAX guard column /30/1,000/1,000 Å (5 μm). The eluent contained 0.1% TFA + 0.1 M NaCl in water. Samples were run at 1 mL min^{-1} at 30 °C. Analyte samples were filtered through a nylon membrane with 0.45 μm pore size prior to injection. P2VP (620-1,160,000) standards were used to calibrate the SEC system. Experimental $M_{n,SEC}$ and $Đ$ (M_w/M_n) values of synthesized polymers were determined using PSS WinGPC UniChrom GPC software.

Flow cytometry. Flow cytometry was conducted on CytoFLEX Beckmann Coulter, Brea, CA, U.S. For each experiment, 20,000 cells per sample were analyzed using bandpass filter 525/40 nm (B525-FITC channel).

Multi-plate reader. PrestoBlue assay and CytoTox-ONE assay were measured with the multi-plate reader Tecan infinite M200Pro, Germany, using settings described in the respective method section. Fluorescence measurements for the AccuBlue® Assay were measured using the plate reader Tecan Spark Control using the wavelength setting described in the method section.

Dynamic Light Scattering (DLS). The hydrodynamic diameters of the nano assemblies were monitored using a Zetasizer Nano ZS (Malvern Instruments, Germany) with a He−Ne laser operating at a wavelength of 633 nm.

Cell counter. For cell culture, fluidlab R-300 (anvajo GmbH, Germany) was used.

Gel photographs: Agarose gels were captured under transillumination at 312 nm using a transilluminator (Intas GmbH, Germany) and the gel documentation system DOC-PRINT CX3 (VILBER, France).

Materials

Acryloyl chloride (97%), triethyl amine (TEA) (99.5%), 4 M HCl, dioxane, sodium acetate trihydrate (NaOAc×3H_2O), 1,8-diazabicyclo[5.4.0]undec-7-ene (DBU) and 4,4-azobis(4-cyanovaleric acid) (ACVA) (98%) were obtained from Sigma-Aldrich (Germany). L-histidine, methyl L-arginate dihydrochloride and *N,N'*-bis(tert-butoxycarbonyl)-1H-pyrazole-1-carboxamidine were obtained from TCI chemicals (Germany). Tert-butyl (4-aminobutyl)carbamate, (97%) was obtained from BLDpharm (Germany). Dialysis membrane Spectra/Por 6 MWCO 2000, 38 mm were purchased from CarlRoth (Germany). Acetic acid glacial (HOAc), sodium carbonate (Na_2CO_3), sodium hydrogen carbonate ($NaHCO_3$), sodium hydroxide (NaOH) and sodium chloride (NaCl) were obtained from Fisher Scientific. The chain transfer agent, 2 (butylthiocarbonothioylthio) propanoic acid (PABTC) was prepared inhouse following a previously reported procedure.[1] Methanol, chloroform, tetrahydrofuran, dichloromethane, ethyl acetate, dioxane and hexane were distilled on site.

For biological investigations all the following materials were ordered from the suppliers stated in brackets: 8-well chamber slides (ibidi, Germany), TC treated cell culture flasks (Greiner Bio-One International GmbH and Labsolute, Th. Geyer GmbH & Co. KG, Germany), TC treated multi-well cell culture plates (VWR International GmbH, Germany), L929 cells (CLS, No. 400260 Cell Lines Service GmbH, Germany) HEK293T (DSMZ, No. ACC635, Germany), Dulbecco's Modified Eagle's Medium (DMEM) and 4-(2-hydroxyethyl)-1-piperazineethanesulfonic acid (HEPES) buffer 1M, phosphate-buffered saline (PBS), fetal bovine serum (FBS), trypsin-EDTA and penicillin-streptomycin were purchased from Capricorn Scientific GmbH, Germany. PrestoBlue™ cell viability reagent (Thermo Fisher Scientific, Germany), CytoTox-ONE™ Homogeneous Membrane Integrity Assay (Promega, Germany), linear poly(ethylene imine) (LPEI, 25 kDa, Polysciences, Germany). pDNA encoding the enhanced green fluorescent protein (EGFP) for transfection studies was isolated with the EndoFree Mega Plasmid Kit (Qiagen, Germany) from E. coli containing pEGFP-N1 (4.7 kb, Clontech, USA), pKMyc was a gift from Ian Macara (Addgene plasmid #19400;http://n2t.net/addgene: 19400;RRID: Addgene_19400). Addgene_19400), Hoechst (Invitrogen), Calcein and Hanks' Balanced Salt Solution (Sigma Aldrich).

For binding experiments, the following materials and chemicals were used: ethylenediaminetetraacetic acid disodium salt (EDTA) and glacial acetic acid were obtained from Sigma-Aldrich (USA). Tris(hydroxymethyl) aminomethane (TRIS) and glycerol 85% were purchased from CarlRoth. GelRed®, AccuBlue® High Sensitivity dsDNA Quantitation Kit (both Biotium, Hayward, USA) and peqGOLD agarose unviversal were ordered at VWR (Germany).

Synthesis and Characterization.

Monomers

Methyl acryloyl-L-argininate hydrochloride (ArgOMe): The procedure was adopted from literature and modified.[2, 3] Methyl L-argininate dihydrochloride (17.60 g, 63.53 x 10^{-3} moles) was weighed into a flask and dissolved in water (20 mL). After, a 150 mL aqueous solution of dissolved sodium carbonate (17.60 g, 165.73 x 10^{-3} moles) was added. The mixture was cooled in an ice bath under argon for 15 min. Acryloyl chloride (4.5 mL, 55.24 x 10^{-3} moles) was then added dropwise with a syringe pump, over approx. 1 h. After addition, the ice bath was removed after 30 min and the reaction was left for 2 h, then the water was removed under vacuo. The crude product was dried on celite overnight. The celite was split into 3 portions and each was purified using a dry column vacuum chromatography. First, tetrahydrofuran was flushed through until the top spot on the TLC was done eluting. Then, methanol (MeOH): chloroform ($CHCl_3$) (1:1.5) was used until the monomer was done eluting. The fractions were collected and dried under vacuo yielding yellow solid (7.1 g, 46% yield). HRMS (ESI) m/z: $[C_{10}H_{19}N_4O_3]^+$ calcd, 243.1452; found, 243.1449.

Acryloy-L-histidine (His): The procedure was adopted from literature and modified.[4, 5] L-histidine (10 g, 64.45 x 10^{-3} moles) was dissolved in a freshly made 4 M NaOH solution (37 mL) and cooled in an ice bath under argon for 15 min. Acryloyl chloride (6.0 mL, 74.12 x 10^{-3} moles) was then added dropwise with a syringe pump over approx. 1 h. After addition, the ice bath was removed after 30 min and the reaction was left for 2 h, then the water was removed under vacuo. The crude product was mixed with celite and dried overnight. The celite was split into 3 portions and each fraction was purified using a dry column vacuum chromatography. First, a mixture of MeOH: $CHCl_3$ (1:1) was used until the top spot on the TLC was done eluting. Then MeOH: ethyl acetate (EtOAc) (2:1) was used until the monomer was done eluting. The fractions were collected and dried under vacuo yielding yellow solid (8.5 g, 57% yield). HRMS (ESI) m/z: $[C_9H_{10}N_3O_3]^-$ calcd, 208.0728; found, 208.0737.

N-(4-guanidinobutyl) acrylamide hydrochloride (GBAm): *Step 1*: The procedure was adopted from literature and modified.[6] *tert*-Butyl (4-aminobutyl)carbamate (12 g, 63.74 x 10^{-3} moles) and triethyl amine (10.7 mL, 76.49 x 10^{-3} moles) were added to a flask and dissolved in dichloromethane (DCM) (500mL). The mixture was cooled in an ice bath and purged with argon. Acryloyl chloride (6.2 mL, 76.49 x 10^{-3} moles) was added dropwise using a syringe pump. After addition, the ice bath was removed after 30 min and the reaction was left stirring at RT for 4 h. The reaction was first washed with water (500 mL x 4), followed by $NaHCO_3$ (300 mL x 3) and then brine 100 mL once. The product solution was dried over $MgSO_4$, concentrated under vacuo and isolated with column chromatography (EtOAc: hexane, 2:1) (Yield: 69%). Afterwards, the product, tert-butyl (4-acrylamidobutyl)

carbamate (12 g, 49.52 x 10^{-3} moles) was deprotected by dissolving it in DCM (10 mL) in a 50 mL falcon tube. 4 M HCl in dioxane (anhydrous) (35 mL) was added and the mixture closed with a perforated lid and left stirring for 5 h. Afterwards the solution was precipitated with cold diethyl ether and centrifuged. The solvent mixture was decanted off and the precipitation repeated 2 more times, redissolving in methanol. The product was then dried under vacuo (yield: 87%). *Step 2*: 4-acrylamidobutan-1-aminium chloride (5.0 g, 27.99 x 10^{-3} moles) and 1,8-diazabicyclo[5.4.0]undec-7-ene (9.1 mL, 60.73 x 10^{-3} moles) were dissolved in DCM (150 mL) and cooled in an ice bath. *N,N'*-bis(tert-butoxycarbonyl)-1H-pyrazole-1-carboxamidine (10.0 g, 32.18 x 10^{-3} moles) was dissolved in DCM (30 mL) and added dropwise to the cooled solution with a dropper funnel. The reaction mixture was left stirring overnight at RT. Next, the mixture was washed with water (300 mL x 4) and concentrated under vacuo. The concentrated mixture was isolated with column chromatography using EtOAc: MeOH (20:1) (yield: 73%). Afterwards, the product (8.0 g, 20.81 x 10^{-3} moles) was deprotected as described in step 1, using DCM (10 mL) and 4 M HCl in dioxane (anhydrous) (26 mL). The product was then dried under vacuo (yield: 81%). HRMS (ESI) m/z: $[C_8H_{17}N_4O]^+$ calcd, 185.1397; found, 185.1391.

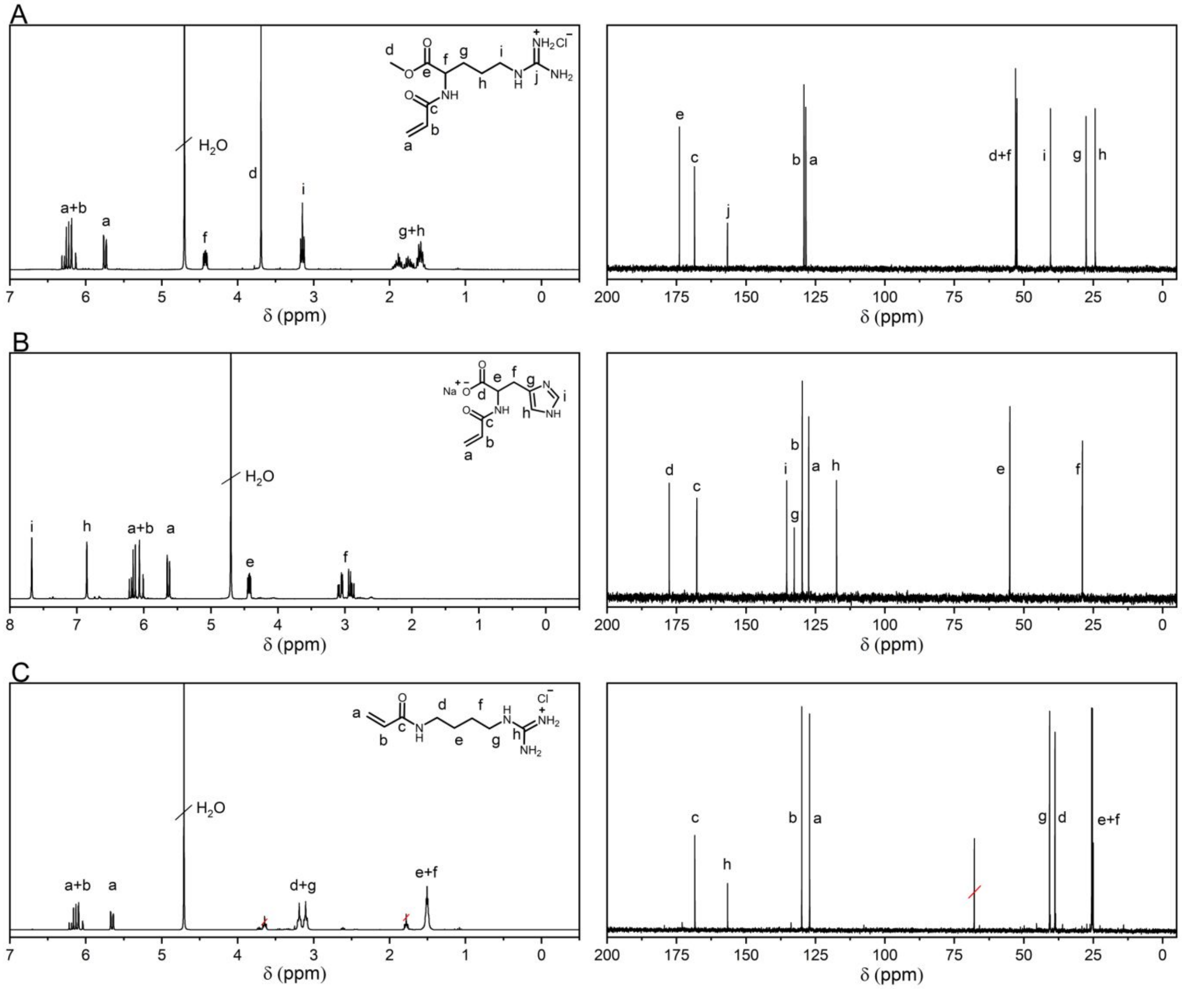

Figure S1. Summary of ^{1}H NMR (300 MHz) and ^{13}C NMR (75 MHz) of each monomer, ArgOMe (A), His (B) and GBAm (C), using D_2O as a solvent. GBAm contained THF residues (peaks crossed in red).

Polymers

Calculations for RAFT Polymerization.

Monomer conversion (p) was calculated from ^{1}H NMR data by comparing the integrals of vinyl peaks (5.5-6.6 ppm) against an external reference (1,3,5-trioxane, ~5.2 ppm) before (t = 0) and after (t = final) polymerization. The theoretical number-average molar mass ($M_{n,th}$) was then calculated using Equation (S1):

$$M_{n,th}\left(\frac{g}{mol}\right) = (Mw_{His} * DP * p) + \left(Mw_{ArgOMe/GBAm} * DP * p\right) + Mw_{PABTC} \quad \text{……. (Equation S1)}$$

Where $DP = DP_{target} * p$ for each monomer, Mw_{monomer} and Mw_{CTA} are the molecular weight of the monomers, and PABTC, respectively.

Kinetics

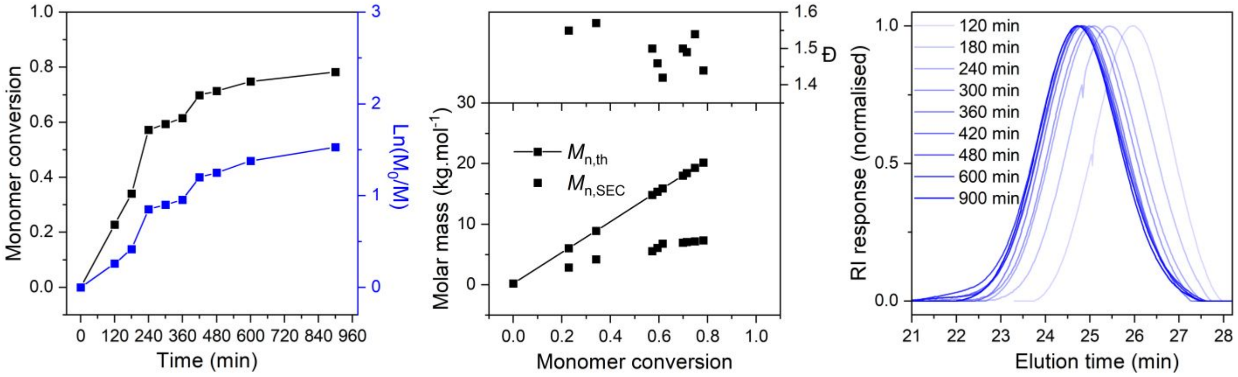

Figure S2. Summary of polymerization kinetics. The left plot illustrates monomer conversion over time (determined by ^{1}H NMR), middle plot shows the comparison of calculated and measured molar masses at different monomer conversions, and the right plot shows SEC traces (Water + 0.1% TFA and 0.1 M NaCl with P2VP calibration).

(Propionic acid)yl butyl trithiocarbonate (PABTC) (10.0 mg, 4.19 × 10^{-5} moles), methyl acryloylargininate (ArgOMe) (0.58 g, 2.10 × 10^{-3} moles), *N*-acryloyl-histidine (His) (0.49 g, 2.10 × 10^{-3} moles), a 2.6 mL 7:3 v/v % premade solution of acetate buffer (1 M, adjusted to pH 5) and dioxane were respectively added to an 8 mL microwave vial equipped with a magnetic stirring bar. Additionally, 1 equivalent of acetic acid (120 μL) to His, a 0.5 wt.% solution of 4,4′-azobis(4-cyanovaleric acid) (ACVA) in dioxane (448.0 mg, 2.24 mg ACVA, 7.99 × 10^{-6} moles) and 1,3,5-trioxane (external NMR standard, 29 mg) were introduced to a 8 mL microwave vial equipped with a magnetic stirring bar. The vial was sealed, and the solution deoxygenated by bubbling argon through it for 10 min. The vial was placed in an oil bath at 70 °C and allowed to stir for 15 h. Kinetic points were taken prior to start of the reaction and then hourly for 8 h, then at 10 h and lastly, 15 h. ^{1}H NMR was used to calculate conversion and theoretical number-average molar mass ($M_{n,th}$) using aforementioned

formulas (Equation S1). Aqueous cationic-SEC was used to determine molar mass distributions (Figure S2). *Purification*: After the reaction, 2M HCl was added to the polymer solution until approx. pH 3. The solution was added to a dialysis tubing, pre-wetted RC Spectra/Por® 6; 2 kD MWCO and dialyzed in pH 3 adjusted deionized water (using 0.2 M HCl). This was done over 4 days, changing the water at least 3 times a day. The polymer was then dried under reduced pressure and lyophilized to give a solid.

Synthesis of P[(ArgOMe)-*co*-(His)] (ArgOMe-His$_{35/44/66/98}$) and P[(GBAm)$_{99}$-*co*-(His)$_{99}$] (GBAm-His$_{99}$)

The polymer library was synthesized following the kinetics and purification protocol (added quantities are provided in Table S1). For each polymer, 1,3,5-trioxane (28-32 mg) and a premade solution of acetate buffer (1 M, adjusted to pH 5) and dioxane in a 7:3 v/v ratio were used. Samples were taken prior to starting the reaction and at the end, to determine conversion. After purification, each polymer was characterized by ^{1}H NMR and aqueous cationic-SEC as shown in Figure S3.

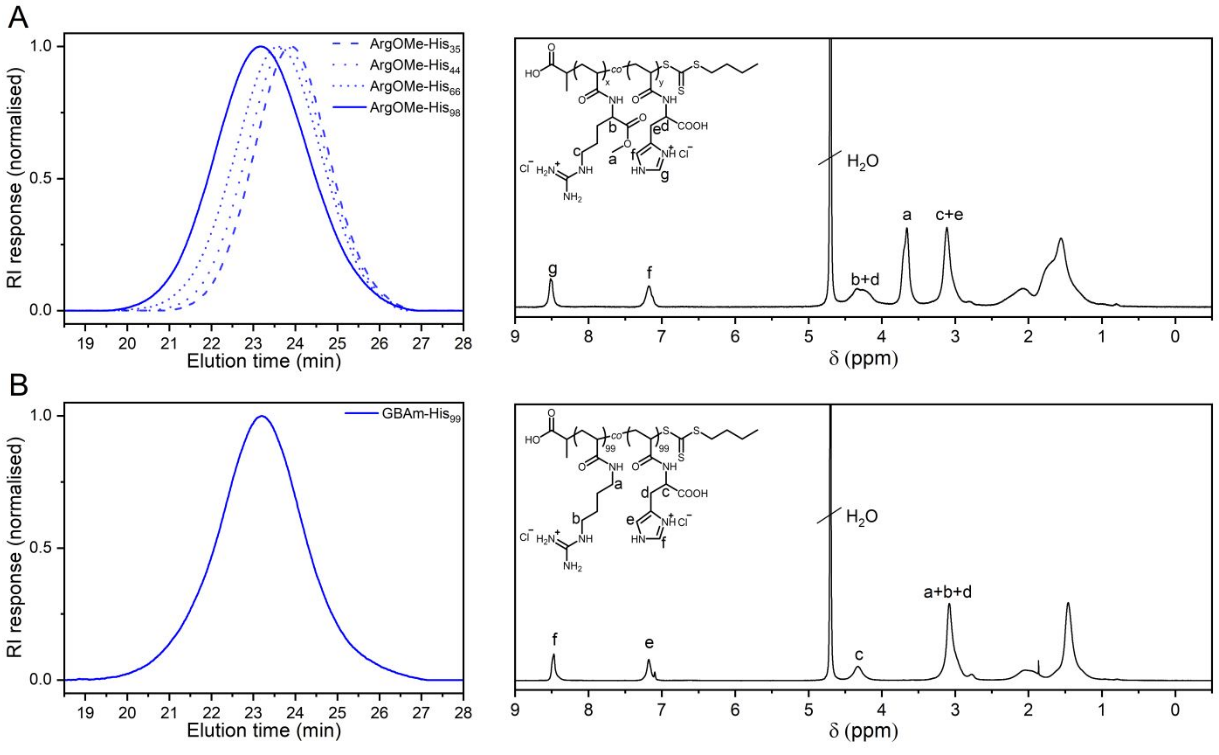

Figure S3. SEC plots and typical ^{1}H NMR (in D_2O) of **ArgOMe-His$_{35/44/66/98}$** library (A) and **GBAm-His$_{99}$** (B), respectively.

Synthesis of P(ArgOMe)$_{99}$

The polymer was synthesized following the kinetics and purification protocol. For each polymer, 1,3,5-trioxane (25 mg) and a 6.4 mL premade solution of acetate buffer (1 M, adjusted to pH 5) and dioxane in a 1.2:1 v/v ratio was used. Samples were taken prior to starting the reaction and at the end, to determine conversion (added quantities are provided in Table S2). After purification, the polymer was characterized by ^{1}H NMR and aqueous cationic-SEC as shown in Figure S4.

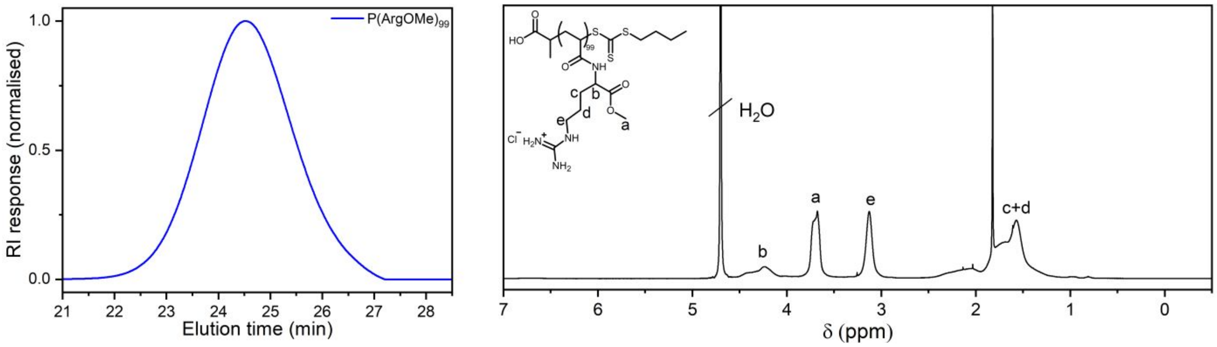

Figure S4. SEC plot and ^{1}H NMR (in D_2O) of **P(ArgOMe)$_{99}$**.

Synthesis of P(His)$_{83}$

The polymer was synthesized following the kinetics and purification protocol. However, an adjustment was made for the purification, i.e., deionized water was used instead of the acidic aqueous solution, but the rest was perfomed the same way. For each polymer, 1,3,5-trioxane (32 mg) and a 5.7 mL premade solution of deionized water and dioxane in a 3:1 v/v ratio was used. Samples were taken prior to starting the reaction and at the end, to determine conversion (added quantities are provided in Table S2). After purification, each polymer was characterized by ^{1}H NMR and aqueous cationic-SEC as shown in Figure S5.

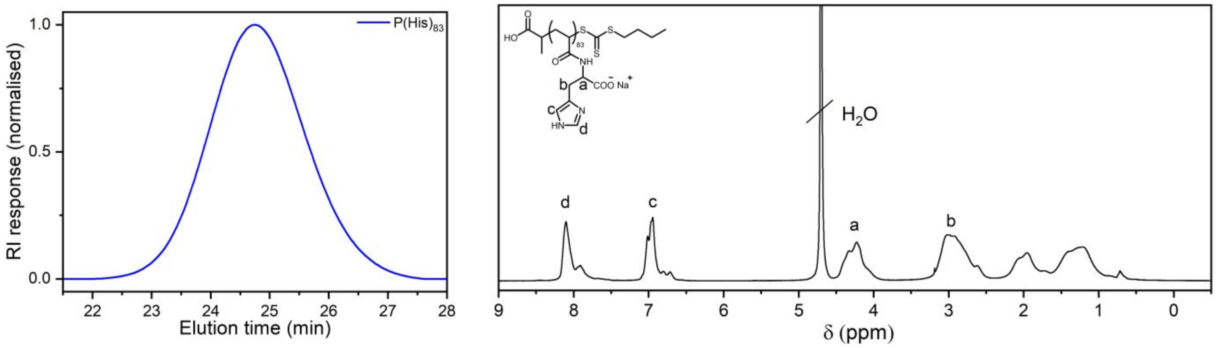

Figure S5. SEC plot and ^{1}H NMR (in D_2O) of **P(His)$_{83}$**.

Synthesis of P(GBAm)$_{88}$ and P[(GBAm)$_{102}$-*co*-(His)$_{51}$] (GBAm-His$_{51}$)

P(GBAm)$_{88}$: PABTC (5.7 mg, 2.39 × 10^{-5} moles), GBAm (0.56 g, 2.53 × 10^{-3} moles), a 4.5 mL 7:3 v/v % premade solution of acetate buffer (1 M, adjusted to pH 5): dioxane, a 0.5 wt.% solution of ACVA in dioxane (129.2 mg, 0.65 mg ACVA, 2.30 × 10^{-6} moles) and 1,3,5-trioxane (26.3 mg) were introduced to a 8 mL microwave vial equipped with a magnetic stirring bar. GBAm-His$_{51}$: PABTC (5.7 mg, 2.39 × 10^{-5} moles), GBAm (0.56 g, 2.53 × 10^{-3} moles), His (0.29 g, 1.27 × 10^{-3} moles), a 3.0 mL 7:3 v/v % premade solution of acetate buffer (1 M, adjusted to pH 5): dioxane, 1 eq acetic acid (140 µL) to His, a 0.5 wt.% solution of ACVA in dioxane (204.3 mg, 1.02 mg ACVA, 3.64 × 10^{-6} moles) and 1,3,5-trioxane (33 mg) were introduced to a 8 mL microwave vial equipped with a magnetic stirring bar. Each vial was sealed, and the solution deoxygenated by bubbling argon through it for 10 min. The vials were placed in an oil bath at 70 °C and allowed to stir for 15 h. Conversion was monitored by ^{1}H NMR, taking samples prior to reaction start and at the end. After the reaction, 2M HCl was added to the polymer solution until approx. pH 3. The solutions were dialysed in DI water using a tubing pre-wetted RC Spectra/Por® 6; 2 kD MWCO, for 2 days, changing the water at least 3 times a day. After, the polymers were lyophilized (Figure S6).

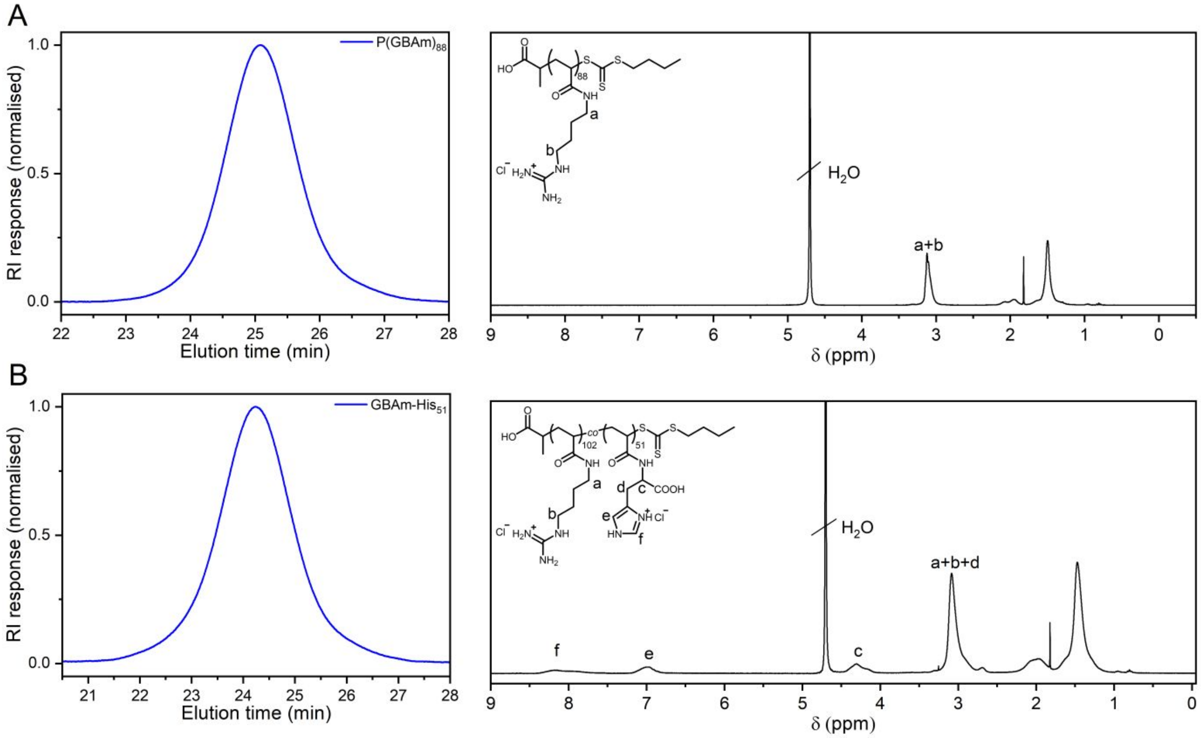

Figure S6. SEC plot and ^{1}H NMR (in D_2O) of P(GBAm)$_{88}$ (A) and GBAm-His$_{51}$. P(GBAm)$_{88}$: $\mathbf{M_{n,th}}$**:** 19.6 kg mol^{-1}, $\mathbf{M_{n,SEC}}$**:** 5.5 kg mol^{-1}, **Đ:** 1.3. GBAm-His$_{51}$: $\mathbf{M_{n,th}}$**:** 35.2 kg mol^{-1}, $\mathbf{M_{n,SEC}}$**:** 9.2 kg mol^{-1}, **Đ:** 1.5.

Table S1. Summary of polymer synthesis quantities.

	ArgOMe-His$_{35}$	ArgOMe-His$_{44}$	ArgOMe-His$_{66}$	ArgOMe-His$_{98}$	GBAm-His$_{99}$	P(ArgOMe)$_{99}$	P(His)$_{83}$
DP (GBAm)	-	-	-	-	99	-	-
DP (ArgOMe)	105	98	98	98	-	99	-
DP (His)	35	44	66	98	99	-	83
PABTC (mg)	12	12	12	10	10	8.2	12
PABTC (moles)	5.03×10^{-5}	5.03×10^{-5}	5.03×10^{-5}	4.19×10^{-5}	4.19×10^{-5}	3.44×10^{-5}	5.03×10^{-5}
GBAm (g)	-	-	-	-	0.98	-	-
GBAm (moles)	-	-	-	-	4.45×10^{-3}	-	-
ArgOMe (g)	1.49	1.49	1.49	1.24	-	1.02	-
ArgOMe (moles)	5.34×10^{-3}	5.34×10^{-3}	5.34×10^{-3}	4.45×10^{-3}	-	3.65×10^{-3}	-
His (g)	0.41	0.56	0.83	1.03	1.03	-	1.16
His (moles)	1.76×10^{-3}	2.42×10^{-3}	3.57×10^{-3}	4.45×10^{-3}	4.45×10^{-3}	-	5.03×10^{-3}
ACVA 0.5 wt.% (mg)	464	468	466	465	465	186	414
ACVA (moles)	8.28×10^{-6}	8.35×10^{-6}	8.32×10^{-6}	8.32×10^{-6}	8.32×10^{-6}	3.31×10^{-6}	7.38×10^{-6}
Acetic acid (uL)	100	138	205	255	255	-	-
Solvent (uL)	5500	5500	5500	5500	5500	6400	5700
[PABTC]/[ACVA]	6.08	6.03	6.05	5.05	5.05	10.38	6.83
T (°C)	70	70	70	70	70	70	70
Time (min)	900	900	900	900	900	480	330

Titrations

Titration of the polymers was conducted using a Metrohm OMNIS integrated titration system (with dynamic flow rate adjustment). For polymers **ArgOMe-His$_{35/44/66/98}$**, a typical measurement: 100 mg of polymer was dissolved in 10 mL 0.15 M HCl and titrated against 0.15 M NaOH solution. **GBAm-His$_{99}$** (114 mg) was dissolved in 0.2 M HCl (10 ml) and titrated against 0.15 M NaOH. Lastly, **P(His)$_{83}$** (82 mg) was dissolved in 150 mM NaCl (8 mL),

adjusted to pH 10.2 using sat. Na_2CO_3 dropwise and titrated against 0.2 M HCl. The degree of charge (DoC) at different pH values was calculated by Equation S2 as the amount of negatively or positively charged units per total amount of amine (imidazole) groups[7]:

$$R - NH^{+}(CH_2)_x + OH^{-} + H_2O \rightleftharpoons R - N(CH_2)_x + H_3O^{+} + OH^{-}$$

$$\text{DoC (histidine)} = \frac{[R - NH^{+}(CH_2)_x]}{[R - N(CH_2)_x]_{tot}} \cdot 100 \qquad \text{....(Equation S2)}$$

Subsequently, a curve of DoC (y-axis) and pH (x-axis) was plotted. The pK_a values were determined as the pH value where the DoC was 50 % (y = 50).

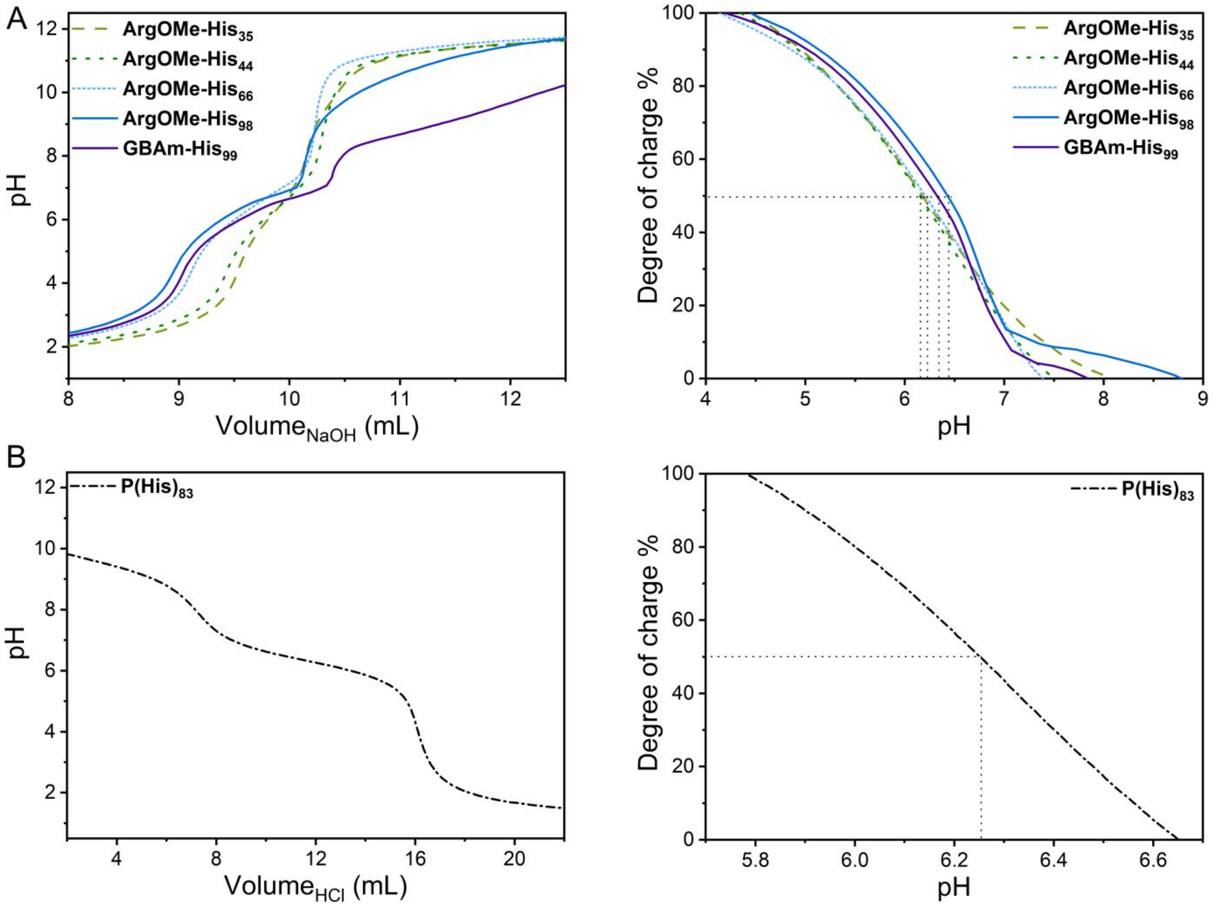

Figure S7. Titration curves of **ArgOMe-His$_{35/44/66/98}$**, **GBAm-His$_{99}$** **(A, left)** and **P(His)$_{83}$** **(B, left)**. The right plots (A and B) show degree of charge (calculated from Equation S2) plotted against the pH, and used to determine the apparent pK_a values of the polymers.

Biological assays

Cell culture: The mouse fibroblast cell line L929 and human embryonic kidney cell line HEK293T were cultured in Dulbecco's Modified Eagle's Medium (DMEM, 1 g L^{-1} glucose), supplemented with 10 % (v/v) fetal bovine serum (FBS), 100 U mL^{-1} penicillin, and 100 μg mL^{-1} streptomycin (D10) at 37 °C in a humidified 5% (v/v) CO_2 atmosphere. One day before the PrestoBlue assay, L929 cells were seeded in a 96 well-plate at a cell concentration of 0.1 x 10^6 cells mL^{-1} in a total volume 100 μL D10 per well without using the outer wells. 24 h before transfection efficiency studies, HEK293T cells were seeded in a 24 well-plate at a cell concentration of 0.2 x 10^6 cells mL^{-1} in 500 μL D10, supplemented with 10 mM HEPES (D10H). Cells were preincubated for 24 h to adhere and to reach a cell confluency > 70%. One hour before the experiment started, the medium was changed to fresh D10H. To count cells for cell seeding fluidlab R-300 anvajo was used.

N*/P ratio calculations

The N*/P ratio was calculated according to a previously published protocol.[8] The N*/P ratio was defined as the ratio of the total amount of protonable guanidinium/LPEI amine groups in polymer solution in relation to the total amount of phosphates in the pDNA solution. The volume of polymer needed to prepare polyplexes with 30 μg mL^{-1} pDNA at different N*/P ratios was calculated as described by the following equations Equation S3:

$$V_{total} \cdot P = V_{poly} \cdot N_{poly}$$

$$V_{poly} = \frac{V_{total} \cdot P}{N_{poly}}$$

$$V_{poly} = V_{total} \cdot \frac{n_{pDNA} \cdot P}{n_{poly} \cdot N}$$

$$V_{poly} = V_{total} \cdot \frac{m_{pDNA} \cdot P \cdot M_{poly}}{m_{poly} \cdot N \cdot M_{pDNA}} \qquad \text{(Equation S3)}$$

Where V_{total}, P, V_{poly} and N_{poly} are the total required volume, the total number of phosphates of the pDNA, the required volume of polymer and the total number of protonable amines of the polymer, respectively.

Polyplexation: Plasmid DNA (pDNA) was diluted in 5% glucose supplemented with 20 mM HEPES buffer (HBG, pH 7.4) to have a master mix with a pDNA concentration, which was twice as high as in the final polyplex solution. The polymers were diluted in 20 mM sodium acetate buffer (NaOAc buffer, pH 5.4) at double the concentration as aimed for in the final N*/P ratio. This ratio was

calculated using Equation S3. The master mix was added 1:1 (v/v) to the diluted polymer solution. Immediately the mixture was vortexed for 10 s at maximum speed and further incubated for 15 min at room temperature.

Interaction of polymers and genetic material: To determine the ability of the polymers to complex pDNA, horizontal gel electrophoresis was used. Polyplexes were prepared and mixed with loading buffer (1 mM ethylenediaminetetraacetic acid disodium salt (EDTA), 40 mM tris(hydroxymethyl) aminomethane (TRIS), 50% (v/v) glycerol (85%). Free DNA and free polymer in the same amount as for N*/P 20 served as controls. All samples were loaded on a 1% agarose gel containing GelRed®. The horizontal electrophoretic separation was performed at 80 V for 1 h in TAE buffer (40 mM TRIS,1 mM EDTA and 0.1% acetic acid). Gel photographs were analyzed using the software BioVision (VILBER, Collegien, France) after capturing the gel photographs under UV transillumination at 312 nm. Additionally, the percentage amount of non-complexed pDNA was quantified using a dye exclusion assay (AccuBlue® High Sensitivity dsDNA Quantitation Kit). Polyplex samples containing 100 ng pDNA were pipetted in a 96 well plate in triplicates and mixed with 200 µL working solution according to the manufacturer's protocol (mixture of 100X enhancer and quantification buffer in a ratio of 1:100). After shaking at 200 rpm in the dark for 10 min, fluorescence intensity was determined using the Tecan Spark Control (λ_{Ex} = 485 nm, λ_{Em} = 530 nm). Free polymer without pDNA was used as negative control, the buffer used for polyplexation as blank and free pDNA without polymer was set as 100% unbound pDNA. Results were calculated using the following equation:

$$\text{Free pDNA/ \%} = \frac{F_{Sample}-F_{blank}}{F_{free\ DNA}-F_{blank}} \quad \text{(Equation S4)}$$

For gel electrophoresis and fluorophore exclusion assay, a polyplex sample with linear poly(ethylene imine) (LPEI) at N*/P 20 served as positive control.

Size determination via dynamic light scattering: The hydrodynamic size of the polyplexes was determined with DLS Zetasizer Nano ZS. Each sample was measured at 25 °C after an equilibration time of 30 s. 15 size runs were performed with 0.839 s per run. The counts were detected at an angle of 173°. Water was used as a dispersant with a viscosity of 0.8872 mPa*s (at 25 °C) and a refractive index of 1.33. The mean particle size was approximated as the effective (z-average) diameter and the distribution width as the polydispersity index of the particles (PDI). General purpose was used as an

analysis model assuming a spherical shape of the polyplexes. Data was analyzed using ZS Xplorer software. Results are shown in Figure S8 and Table S2.

Hydrodynamic size determination of polyplexes

Table S2. DLS measurements of polyplexes at physiological pH, with storage at 4 °C over 40 days. t = 0 d was measured three times (n = 3).

Polymer ID	Z-Average / nm			PDI		
	t = 0 d	t = 10 d	t = 40 d	t = 0 d	t = 10 d	t = 40 d
**ArgOMe-His$_{35}$**	61.8 ± 5.6	72.8	66.3	0.15 ± 0.01	0.16	0.21
**ArgOMe-His$_{44}$**	63.8 ± 1.8	55.7	59.7	0.15 ± 0.02	0.14	0.14
**ArgOMe-His$_{66}$**	82.6 ± 24.6	65.2	78.5	0.13 ± 0.02	0.12	0.14
**ArgOMe-His$_{98}$**	102.4 ± 11	97.6	103.6	0.12 ± 0.004	0.11	0.05
**GBAm-His$_{99}$**	83.5 ± 5.7	89.2	90.9	0.2 ± 0.03	0.10	0.23
**P(ArgOMe)$_{99}$**	54.6 ± 4.23	43.4	59.2	0.2 ± 0.03	0.18	0.13
**GBAm-His$_{51}$**	70.17 ± 1.25			0.25 ± 0.01		
**P(GBAm)$_{88}$**	60.38 ± 0.78			0.24 ± 0.004		

Table S3. DLS measurements of polyplexes over time at pH 5.4.

**Polymer ID**	**GBAm-His$_{99}$**		**P(ArgOMe)$_{99}$**	
time	z-Average (nm)	PDI	z-Average (nm)	PDI
0 h	68.9	0.24	62.0	0.31
1 h	70.1	0.23	57.9	0.28
2 h	67.7	0.22	67.2	0.38
3 h	70.7	0.24	71.7	0.37
6 h	69.3	0.23	89.9	0.35

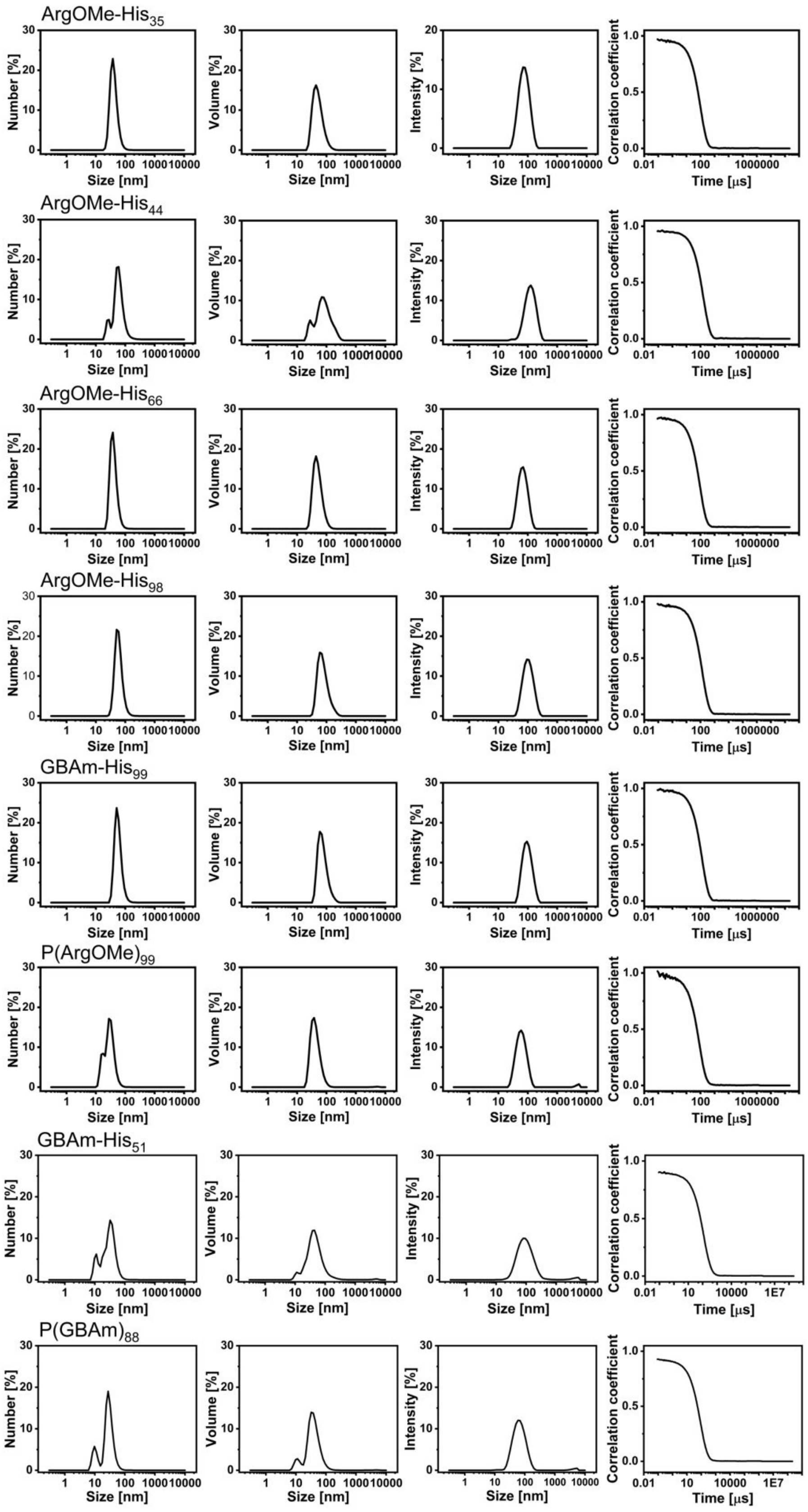

Figure S8. DLS measurement of polyplexes at N*/P 15 at t = 0 d of the first measurement.

Cytotoxicity assays

Cytocompatibility of polymers (PrestoBlue assay): Cytotoxicity of the polymers was assessed by performing a PrestoBlue assay with the L929 cells as recommended by the ISO10993-5 to evaluate the metabolic activity of cells.[9] One hour before treatment, the medium was changed to 90 µL fresh D10H. In triplicate, cells were treated with 10 µL polymers of different dilutions. The polymers were diluted in 20 mM sodium acetate buffer. The tested polymer concentration ranges from 13 to 400 µg mL^{-1}. LPEI was used as control at concentrations of 3 to 100 µg mL^{-1}. Cells were incubated with polymer solution for 24 h. Afterwards the medium was replaced by a 10% (v/v) PrestoBlue solution in a fresh D10, prepared according to the manufacturer's instructions. Cells were further incubated at 37 °C for 45 min and the fluorescence was measured with the multi-plate reader at λ_{Ex} = 570 / λ_{Em} = 610 nm. Control cells treated with buffer on the same plate were defined as 100% viability. Values lower than 70% viability were regarded as cytotoxic. The relative number of viable cells was calculated as follows:

$$\text{Rel. viability / \%} = \frac{FI_{Sample} - FI_0}{FI_{Ctl} - FI_0} \cdot 100 \quad \text{(Equation S5)}$$

Where FI_{Sample}, FI_0, and FI_{Ctrl} represent the fluorescence intensity of a given sample, medium without cells (the blank), and buffer-treated control (100% viability), respectively.

Cytocompatibility of polyplexes (CytoTox-ONE assay): To determine the membrane integrity of polyplex-treated HEK293T cells over 24 h incubation, CytoTox-ONE assay was performed in combination with transfection efficiency assay. After 24 h incubation with the polyplexes (transfection efficiency assay) aliquot of the supernatant was transferred to a 96-well plate in triplicate, followed by equilibration at room temperature for 20 min. Subsequently, 50 µL of CytoTox-ONE reagent was added to each well and incubated for 10 min at room temperature. To stop the reaction, 25 µL of stop solution was added to each well. Cells treated with lysis solution were used as 100% control. Values lower than 90% viability were regarded as cytotoxic. The fluorescence intensity was measured at λ_{Ex} = 570 / λ_{Em} = 610 nm and cytotoxicity was calculated as follows:

$$\text{Rel. cytotoxicity/ \%} = \frac{FI_{Sample} - FI_0}{FI_{Ctrl} - FI_0} \quad \text{(Equation S6)}$$

$$\text{Viability/ \%} = 1 - \text{rel. cytotoxicity} \quad \text{(Equation S7)}$$

Where FI_{Sample}, FI_0, and FI_{Ctrl} represent the fluorescence intensity of a given sample, medium without cells (the blank), and lysis-treated cells (100% cytotoxicity), respectively.

Transfection efficiency

Transfection efficiency: HEK293T cells were seeded as described. One hour before the experiment started, the old cell culture medium was changed to 450 µL fresh D10H and 50 µL polyplex at N*/P 15 and a final pDNA concentration of 3, 2, 1.5, or 1 µg mL^{-1} was added to cells. As positive control for the assay LPEI was used at N*/P 20, which is the optimal transfection condition for LPEI to perform as control from our previous work.[10] In a master mix, cells were treated with 3 µg mL^{-1} of mEGFP-N1 pDNA and control were cells treated with 20 mM HEPES buffer (pH7.4) and 20 mM acetate buffer (pH 5.4). The assay was conducted over 24 h without medium change. For harvesting, cells were treated with 150 µL of Trypsin EDTA and incubated for 10 min at 37 °C (5% CO_2). To stop trypsinization, 350 µL of fresh D10 was added and 250 µL of cell suspension was transferred to 96-well plate for following flow cytometry measurement using the FITC channel (λ = 525/40 nm). Viable, single cells with higher fluorescence intensity were gated into the negative control cells, which were treated with pKMyc-polymer complexes. The gating strategy was applied as shown in Figure S9 below. Results are shown in Figure S10 and S11. **Statistics**: Transfection efficiency was performed at least in triplicate. Statistics were calculated using OriginPro2022b software. One-way analysis of variance (ANOVA) following by a Bonferroni's posthoc test was applied. Statistical significance was illustrated as *$p \leq 0.05$, **$p \leq 0.01$, and ***$p < 0.001$.

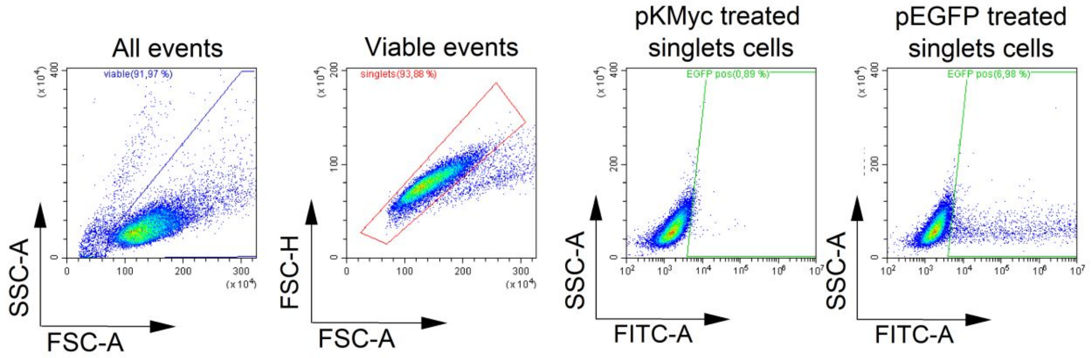

Figure S9. The gating strategy example for transfection (**P(ArgOMe)$_{99}$** N*/P 15, 3 µg mL^{-1} pDNA). Firstly, viable cells were gated according to FSC-A/SSC-A (blue gate) pattern. Followed by gating viable cells in FCS-H/FSC-A (red gate) to distinguish single cells to cell aggregation. Samples treated with pKMyc were gated so that EGFP positive cells in the gate was lower than 1 % (green gate). The EGFP expressing cells were gated using the gate of the pKMyc control.

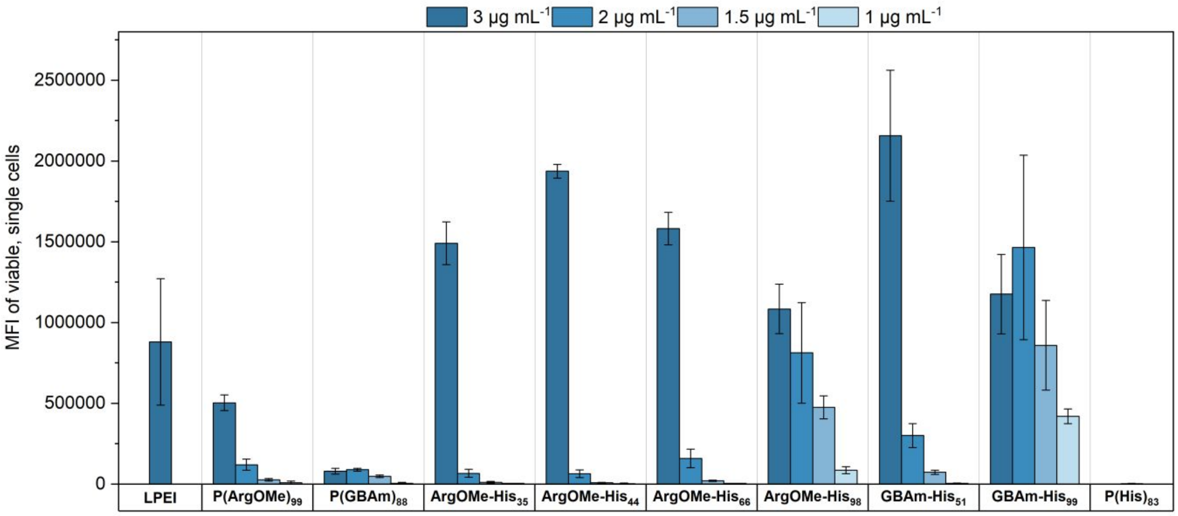

Figure S10. The plot shows the mean fluorescence intensity of viable single cells of a transfection efficiency assay conducted in full growth medium at N*/P 15 with different pDNA concentrations in HEK293T cells over 24 h (n ≥ 3). LPEI at N*/P 20 and 3 µg mL^{-1} of mEGFP-N1 pDNA on cells was used as control.

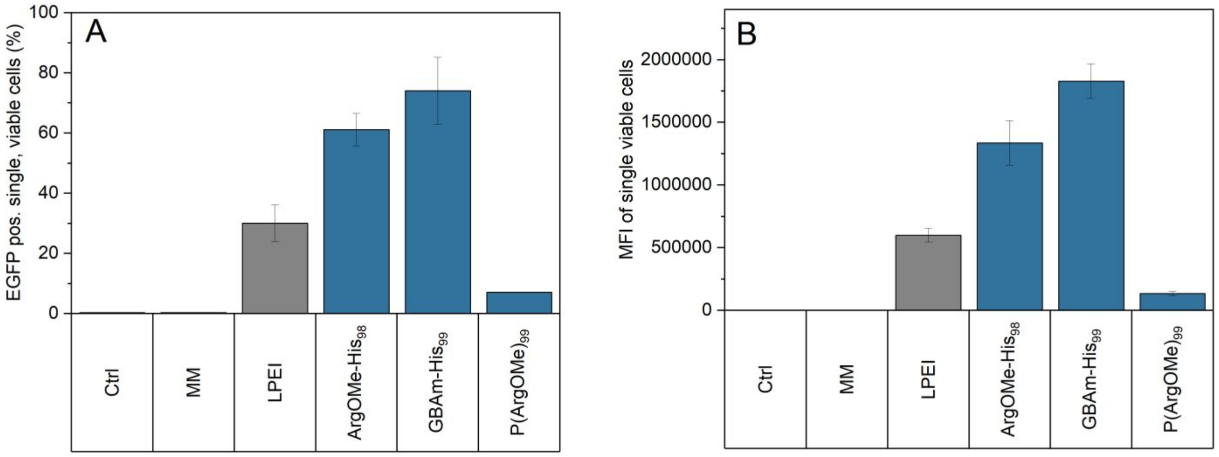

Figure S11. Transfection efficiency assay conducted in full growth medium (D10H) at N*/P 10 with 3 µg mL^{-1} of mEGFP-N1 pDNA in HEK293T cells over 24 h (n ≥ 3). LPEI at N*/P 20 and 3 µg mL^{-1} of mEGFP-N1 pDNA on cells was used as control. Master mix (MM) were cells treated with 3 µg mL^{-1} of mEGFP-N1 pDNA. Ctrl are cells treated with 20 mM HEPES buffer (pH7.4) and 20 mM acetate buffer (pH 5.4). A) Plot shows the transfection efficiency results. B) The plot shows the mean fluorescence intensity of viable single cells.

Endosomal release:

To study the endosomal release HEK293T cells were seeded at 0.2 × 10^6 cells mL^{-1} in D10H in 8-well slides (ibidi, Germany), followed by incubation at 37 °C in a humidified 5% (v/v) CO_2 atmosphere for 24 h. Then cells were simultaneously incubated for 1 h, 4 h, 6 h in D10H with non-permeable dye calcein (25 µg mL^{-1}) and polyplexes (N*/P 15, 3 µg mL^{-1} pDNA). Before imaging, cell nuclei were stained with Hoechst 3334 for 10 min. Following, cells were washed twice with warm Hanks' Balanced Salt Solution, supplemented with 2% FCS and 20 mM HEPES before D10 was added. The live cell imaging was performed using a LSM880, Elyra PS.1 system (Zeiss, Germany). A 40 × 1.4 NA plan apochromat oil objective, argon laser with λ_{Ex} 488 nm (0.3%) and 405 nm (0.5%), emission filters for 410-469 nm (Hoechst) and 490-544 nm (Calcein) with a digital gain of 1 and with a master gain of 700 and a pinhole of 27 µm were used. Non digital offset was used. Images were acquired using the ZEN software, version 2.3 SP1 (Zeiss, Germany).

Image analysis to quantified endosomal release events was performed with ImageJ version 1.54f. First image was separated in different channels. Hoechst and calcein channels were enclosed in the analysis. Appreciate contrast enhancement and auto threshold were applied before binary was made (Process, Binary). For Hoechst image, holes were filled, and radius of the circle was eroded if it is below 5 pixel (Process, Morphology). For calcein, convolution filter was applied with a median with a radius of 10 pixel (Prozess, Filter). Images of both channels were combined by the image calculator ("AND" combination mode), leaving only the nuclei with calcein staining. The calcein release events were counted via "Analyze Particles" (size threshold 30 square micron) and the number of cells (number of nuclei) were counted on the image of the Hoechst channel (size threshold 5 square micron). The percentage of events with calcein release was calculated as follow:

$$\text{Cell with calcein release/ \%} = \frac{\text{Number of nuclei with calcein signal}}{\text{Number of nuclei}} \quad \text{(Equation S8)}$$

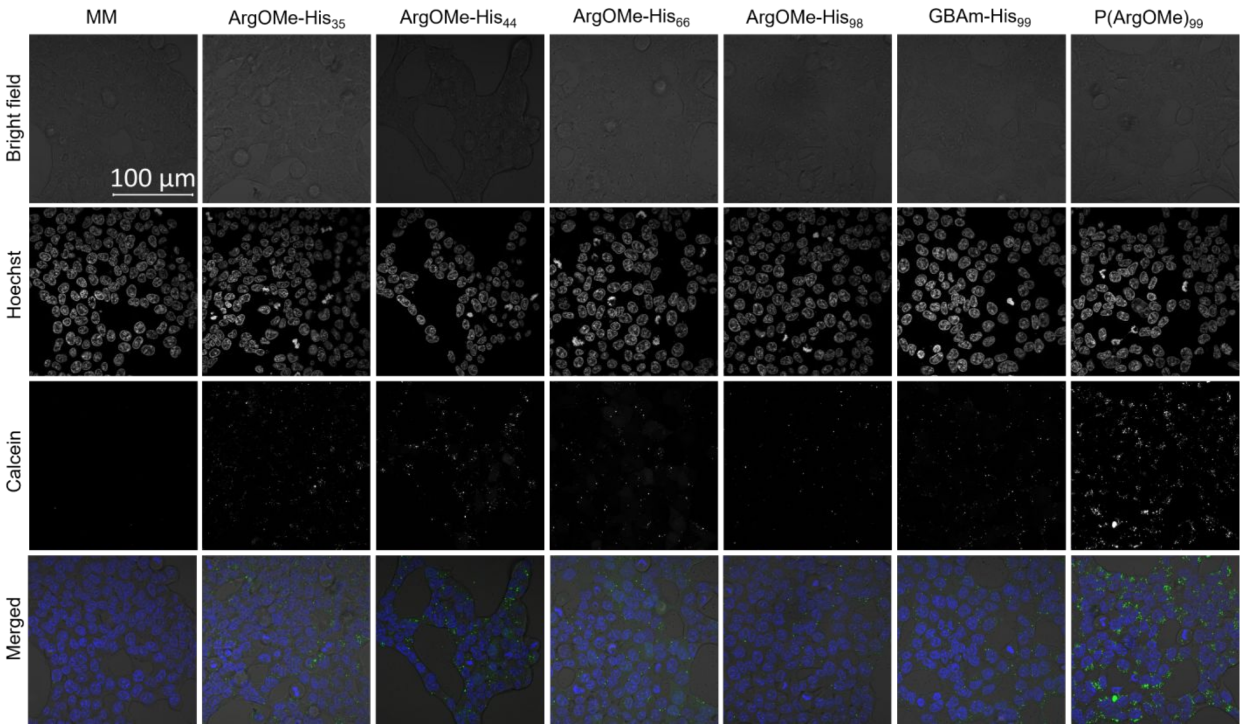

Figure S12. Endosomal release was analyzed via confocal laser scanning microscopy (CLSM) over 1 h in D10H. HEK293T cells were simultaneously incubated with non-permeable dye calcein (25 µg mL^{-1}) and polyplexes (N*/P 15, 3 µg mL^{-1} pDNA). Grey dots indicate endocytotic uptake of calcein within cellular compartments, and diffuse grey fluorescence pattern indicates endosomal calcein release. The cell nuclei were stained with Hoechst 33342. Buffer treated cells and pDNA-master mix-treated cells were used as the controls (Ctrl, MM).

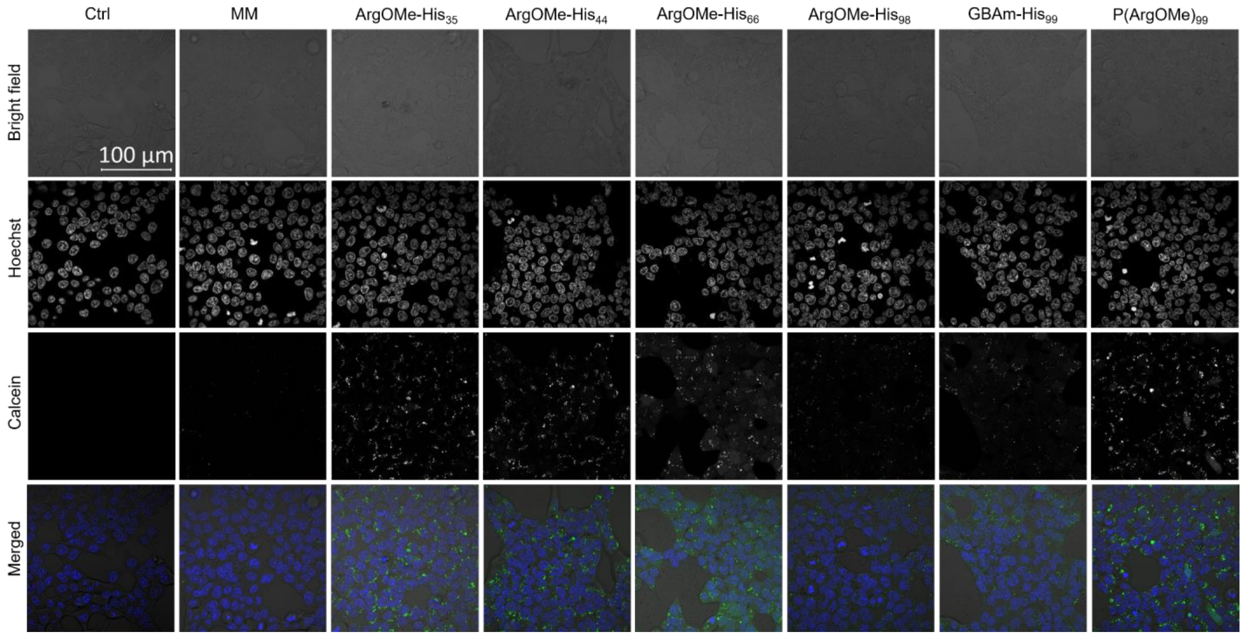

Figure S13. Endosomal release was analyzed via confocal laser scanning microscopy (CLSM) over 4 h in D10H. HEK293T cells were simultaneously incubated with non-permeable dye calcein (25 µg mL^{-1}) and polyplexes (N*/P 15, 3 µg mL^{-1} pDNA). Grey dots indicate endocytotic uptake of calcein within cellular compartments, and

diffuse grey fluorescence pattern indicates endosomal calcein release. The cell nuclei were stained with Hoechst 33342. Buffer treated cells and pDNA-master mix-treated cells were used as the controls (Ctrl, MM).

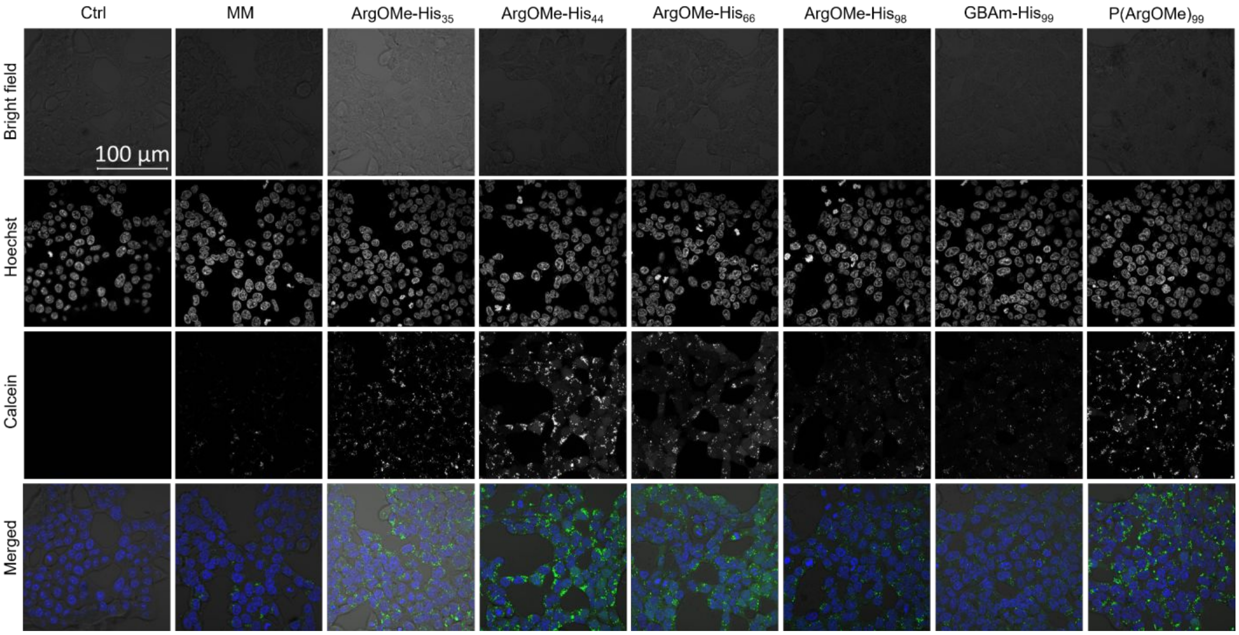

Figure S14. Endosomal release was analyzed via confocal laser scanning microscopy (CLSM) over 6 h in D10H. HEK293T cells were simultaneously incubated with non-permeable dye calcein (25 µg mL^{-1}) and polyplexes (N*/P 15, 3 µg mL^{-1} pDNA). Grey dots indicate endocytotic uptake of calcein within cellular compartments, and diffuse grey fluorescence pattern indicates endosomal calcein release. The cell nuclei were stained with Hoechst 33342. Buffer treated cells and pDNA-master mix-treated cells were used as the controls (Ctrl, MM).

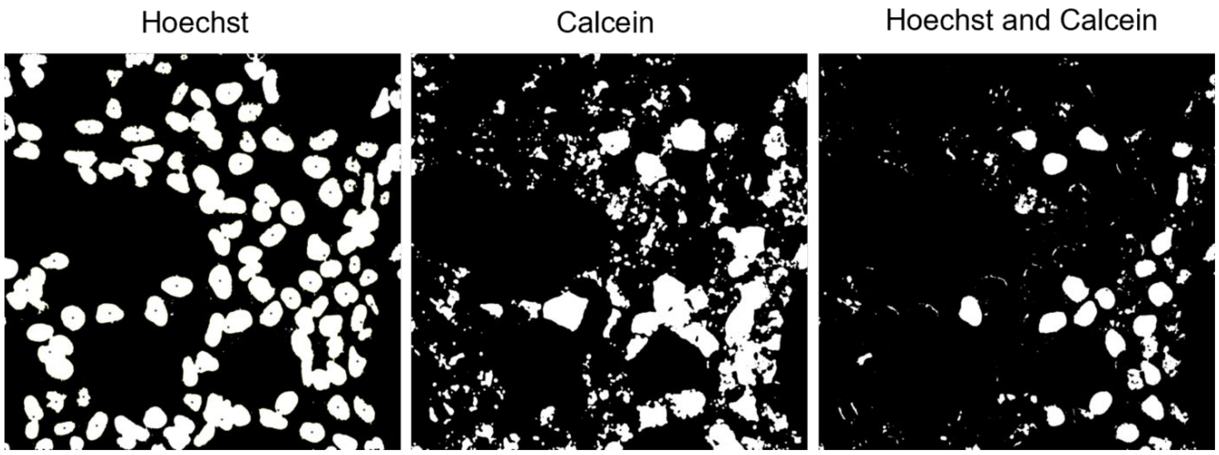

Figure S15. Exemplary images of ArgOMe-His44 after processing with ImageJ to quantify calcein release events after 6 h incubation.

REFERENCES

(1) Larnaudie, S. Supramolecular cyclic peptide-polymer nanotubes as drug delivery vectors. University of Warwick, 2017.
(2) Kim, Y.; Binauld, S.; Stenzel, M. H. Zwitterionic guanidine-based oligomers mimicking cell-penetrating peptides as a nontoxic alternative to cationic polymers to enhance the cellular uptake of micelles. *Biomacromolecules* **2012**, *13* (10), 3418-3426. DOI: 10.1021/bm301351e.
(3) Birnbaum, S. M.; Winitz, M.; Greenstein, J. P. A simplified preparation of d-arginine. *Arch. Biochem. Biophys.* **1956**, *60* (2), 496-498. DOI: 10.1016/0003-9861(56)90455-6.
(4) Bertrand, E.; Gonçalves, C.; Billiet, L.; Gomez, J. P.; Pichon, C.; Cheradame, H.; Midoux, P.; Guégan, P. Histidinylated linear PEI: a new efficient non-toxic polymer for gene transfer. *Chem. Commun.* **2011**, *47* (46), 12547-12549. DOI: 10.1039/C1CC15716G.
(5) Iwakura, Y.; Toda, F.; Suzuki, H. Synthesis of N-[1-(1-substituted 2-oxopropyl)] acrylamides and-methylacrylamides. Isolation and some reactions of intermediates of the Dakin-West reaction. *J. Org. Chem.* **1967**, *32* (2), 440-443. DOI: 10.1021/jo01288a039.
(6) Hobson, L. J.; Feast, W. J. Poly (amidoamine) hyperbranched systems: synthesis, structure and characterization. *Polymer* **1999**, *40* (5), 1279-1297. DOI: 10.1016/S0032-3861(98)00268-7.
(7) Catrouillet, S.; Brendel, J. C.; Larnaudie, S.; Barlow, T.; Jolliffe, K. A.; Perrier, S. Tunable length of cyclic peptide–polymer conjugate self-assemblies in water. *ACS Macro Lett.* **2016**, *5* (10), 1119-1123. DOI: 10.1021/acsmacrolett.6b00586.
(8) Richter, F.; Martin, L.; Leer, K.; Moek, E.; Hausig, F.; Brendel, J. C.; Traeger, A. Tuning of Endosomal Escape and Gene Expression by Functional Groups, Molecular Weight and Transfection Medium: A Structure-Activity Relationship Study. *J. Mater. Chem B.* **2020**. DOI: 10.1039/D0TB00340A.
(9) Standard, I. 10993–5 Biological evaluation of medical devices. *Tests for in vitro cytotoxicity. Geneva, Switzerland: International Organization for Standardization* **2009**, 194.
(10) Solomun, J. I.; Cinar, G.; Mapfumo, P.; Richter, F.; Moek, E.; Hausig, F.; Martin, L.; Hoeppener, S.; Nischang, I.; Traeger, A. Solely aqueous formulation of hydrophobic cationic polymers for efficient gene delivery. *Int. J. Pharm.* **2021**, *593*, 120080. DOI: 10.1016/j.ijpharm.2020.120080.
